# Effectiveness of Psychosocial Interventions for Youth With Mild Intellectual Disabilities or Borderline Intellectual Functioning and Externalising Problems: A Multilevel Meta‐Analysis

**DOI:** 10.1111/jir.70014

**Published:** 2025-07-14

**Authors:** Eva Kühl, Ina M. Koning, Maja Deković, Sander Thomaes, Juliëtte M. Liber

**Affiliations:** ^1^ Department of Psychology Utrecht University Utrecht the Netherlands; ^2^ Department of Educational and Family Studies Vrije Universiteit Amsterdam the Netherlands; ^3^ Department of Clinical Child and Family Studies Utrecht University Utrecht the Netherlands; ^4^ Department of Clinical and Developmental Psychology University of Amsterdam Amsterdam the Netherlands; ^5^ PsyMens Child and Youth Woerden the Netherlands

**Keywords:** borderline intellectual functioning, externalising problems, intellectual disabilities, meta‐analysis, psychosocial intervention

## Abstract

**Objectives:**

This meta‐analysis examined the effectiveness of psychosocial interventions for youth with mild intellectual disabilities or borderline intellectual functioning (MID‐BIF) and externalising problems.

**Methods:**

We assessed 2221 studies for eligibility and included seven studies containing 14 effect sizes. We used a multilevel modelling approach to analyse the data and calculated two different types of effect sizes: one for controlled trials and one for intervention pre‐test to post‐test effects (across all studies).

**Results:**

Both controlled trial effect sizes (*g* = 0.49) and pre‐test to post‐test effect sizes were significant (*g* = 0.53). We were unable to detect heterogeneity of effects because of the small number of eligible studies. The risk of bias was generally high.

**Discussion:**

Results suggest that psychosocial interventions decrease externalising problems among youth with MID‐BIF. Effect sizes appear similar to those for youth without MID‐BIF. We discuss recommendations for how the field can move forward.

## 1 Background

Children and adolescents (hereafter referred to as ‘youth’) with intellectual disabilities, including those with mild intellectual disabilities and borderline intellectual functioning (MID‐BIF; IQ between 55 and 85 and impaired adaptive functioning; American Psychiatric Association 2013), have a higher risk of externalising problems (i.e., defiant, rule‐breaking and aggressive behaviour) compared with typically developing youth (Dekker et al. 2002; Emerson 2003; Peltopuro et al. 2014). Their externalising problems also tend to be more persistent (Emerson et al. 2011), and they are overrepresented in child welfare and justice systems (Slayter and Springer 2011; Thompson and Morris 2016). The high prevalence and severe consequences of externalising problems in youth with MID‐BIF highlight the need for providing evidence‐based psychosocial interventions to decrease these problems.

Psychosocial interventions are nonpharmacological interventions, focused on psychological or social factors (e.g., therapy, prevention or guidance; Burkey et al. 2018). They can effectively reduce and prevent externalising problems among children and adolescents without MID‐BIF, with meta‐analyses showing effect sizes ranging from 0.12 to 0.98 (Baumel et al. 2021; Riise et al. 2021). However, experts assert that youth with MID‐BIF tend to be excluded from existing psychosocial interventions (Van den Bogaard et al. 2020; Wieland et al. 2022) and that research into psychosocial interventions for this population is sparse (Hronis et al. 2017; Kok et al. 2016). Indeed, there are recent examples of intervention studies in which youth with MID‐BIF were excluded (e.g., Goertz‐Dorten et al. 2019; Helander et al. 2018), and treatment protocols for youth regularly include lower IQ thresholds as an exclusion criterion (e.g., Bleumer and Tiemissen 2017; Van Londen et al. 2024; Van Steensel 2023). Experts have previously attributed this to clinicians' beliefs that such interventions are too cognitively demanding (Taylor et al. 2008), although it is unclear to what extent such beliefs still prevail. Regardless, empirical evidence for the effectiveness of psychosocial interventions for youth with MID‐BIF and externalising problems is limited and inconclusive (Kok et al. 2016). We aim to fill this gap through meta‐analysis.

### 1.1 Previous Meta‐Analyses and What Is Still Missing

Previous review studies were sometimes related to the topic of psychosocial interventions for youth with MID‐BIF and externalising problems, but they have not specifically meta‐analysed their effectiveness for this population (e.g., primarily adult populations in Heyvaert et al. 2010, 2012; pharmacological interventions in McQuire et al. 2015; and no meta‐analysis in O'Regan et al. 2022). In a meta‐analysis closely related to this topic, Harvey et al. (2009) examined psychological interventions for children with developmental disabilities and challenging behaviour. The studies in this meta‐analysis most likely included individuals with MID‐BIF and externalising behaviour, but they also included children with a broader range of developmental disabilities (e.g., autism spectrum disorder, intellectual disabilities with an IQ below 55 or multiple disabilities) and a broader range of problem behaviour (i.e., challenging behaviour, including not only aggressive behaviour but also self‐injurious, stereotypical and sexual offending behaviour). The authors concluded that children with aggressive behaviour problems, compared with children who showed other types of challenging behaviour, responded least well to psychosocial interventions. However, they did not report statistical analyses to support this conclusion, nor did they quantify the magnitude of differences in effectiveness between different problem behaviours, such as aggressive behaviours versus stereotypical behaviours.

In a more recent systematic review and meta‐analysis (Kok et al. 2016), the authors investigated the effectiveness of psychosocial interventions for children and adolescents with MID‐BIF. They examined a broad range of problems (i.e., any psychiatric disorder) and found that some promising interventions for children with MID‐BIF were available. However, the authors were unable to differentiate between specific types of disorders or synthesise intervention effects for externalising problems specifically due to a low number of studies. As effect sizes for interventions tend to differ substantially across different problem areas (Weisz et al. 2017), neither the meta‐analysis by Harvey et al. (2009) nor the one by Kok et al. (2016) thus allows for drawing conclusions about the overall effectiveness of psychosocial interventions for children and adolescents with MID‐BIF and externalising problems, specifically.

Since the publication of the meta‐analyses by Harvey et al. (2009) and Kok et al. (2016), important developments have occurred in the field. Experts have called for research into psychosocial interventions for individuals with MID‐BIF and provided recommendations on how to adapt conventional interventions to the needs and abilities of youth with MID‐BIF (Hronis et al. 2017; Taylor et al. 2008). Moreover, a number of trials of adapted psychosocial interventions for individuals with MID‐BIF and various mental health problems have been published (e.g., Hronis et al. 2019; Unwin et al. 2016), including trials involving children and adolescents with externalising problems (e.g., Schuiringa et al. 2017; te Brinke et al. 2022). Given these developments, the first aim of the present meta‐analysis was to synthesise the current evidence base on the effectiveness of psychosocial interventions for reducing externalising problems among youth with MID‐BIF.

### 1.2 Participant, Intervention and Study Characteristics

A second aim of the present study was to investigate potential sources (i.e., characteristics of the included studies) of heterogeneity in intervention effects. Firstly, previous meta‐analyses on externalising problem interventions for youth without MID‐BIF showed that participant characteristics, such as age (e.g., Granski et al. 2020; Riise et al. 2021) and gender (e.g., Granski et al. 2020; Menting et al. 2013), moderate intervention effects. Participants' level of intellectual functioning may also be an important factor to investigate as a potential moderator, given the longstanding assumption that individuals with lower levels of intellectual functioning may benefit less from psychosocial interventions such as psychotherapy (Taylor et al. 2008). Although our study focused on youth with MID‐BIF (i.e., the higher end of intellectual disability IQ ranges), there are differences in social functioning between those in the MID compared to the BIF range (e.g., Van Rest et al. 2020) and differences in intervention effectiveness may thus be expected. Secondly, characteristics of the interventions, such as duration and number of sessions, intervention format (e.g., individual vs. group) or intervention target (e.g., parents vs. children), likely affect the strength of intervention effects. This has been found in previous meta‐analyses on externalising problem interventions for youth without MID‐BIF (e.g., Baumel et al. 2021; Boldrini et al. 2023; Granski et al. 2020; Riise et al. 2021). Thirdly, study characteristics, such as the type of design (control group vs. pre‐post only; e.g., Wilson and Lipsey 2001), the use of intention‐to‐treat analysis (vs. completers only; e.g., Riise et al. 2021) and the risk of bias (i.e., shortcomings in the methodological quality of studies, such as lack of blinding or adequate allocation concealment; e.g., Wood et al. 2008), have been previously identified as moderating factors in meta‐analyses of interventions for individuals without MID‐BIF.

We aimed to describe these participant, intervention and study characteristics. To enhance comprehensiveness, we additionally aimed to describe the level of tailoring to the specific needs of youth with MID‐BIF. The American Association on Intellectual and Developmental Disabilities (AAIDD) describes in their framework of human functioning (Buntinx and Schalock 2010) that individuals with MID‐BIF generally have higher support needs in five areas of functioning: intellectual functioning, adaptive functioning, health, societal participation and the broader context (i.e., they experience more contextual adversities). A high level of care for individuals with MID‐BIF can be obtained when difficulties in each of these five areas are addressed. By reviewing these characteristics, we aim to create a more comprehensive view of the literature, identify potential gaps and provide recommendations.

## 2 Methods

We preregistered our study (PROSPERO ID CRD42023446178) and adhered to PRISMA guidelines (Page et al. 2021). Our study protocol, data and analysis code are available on Open Science Framework: https://doi.org/10.17605/OSF.IO/9RTX7.

### 2.1 Selection Criteria

We included studies and (unpublished) dissertations written in English that sampled youth with MID‐BIF (IQ between 55 and 85 or a MID‐BIF diagnosis), with a mean age of ≤ 18 years old and individual participants' ages of ≤ 23 years old. Studies had to investigate a psychosocial intervention primarily aimed at reducing externalising problems. The intervention could be aimed at any target (e.g., youth, parents and teachers) and implemented in any context (e.g., community‐based, residential settings and schools). We included both controlled (randomised and nonrandomised; any control group) and noncontrolled intervention studies with at least one preintervention and one postintervention measure of externalising problems. We excluded studies that focused on pharmacological interventions exclusively, that sampled participants with severe sensory impairments (hearing, vision) or that had an intervention condition sample size smaller than *n* = 10. See Supporting Information S1 for a detailed description of the eligibility criteria, grouped according to the PICOS acronym.

### 2.2 Literature Search, Selection, and Coding

#### 2.2.1 Search String

We searched PsycINFO, ERIC, MEDLINE, PubMed and EMBASE. We limited our search to peer‐reviewed papers and dissertations in the English language and placed no limit on the publication year. We searched the grey literature database ProQuest for dissertations. We included variations of the following concepts in the search term: ‘intellectual disability’ AND ‘psychosocial intervention’ AND ‘behaviour problems’ AND ‘age’ and included subject headings when possible. The search string was thoroughly piloted in PsycINFO to optimise a balance between sensitivity and precision. See Supporting Information S2 for the full search strings. We completed the first literature search on 21 August 2023. We updated our search on 30 January 2024 but found no additional studies for inclusion.

#### 2.2.2 Study Selection and Coding

**FIGURE 1:**
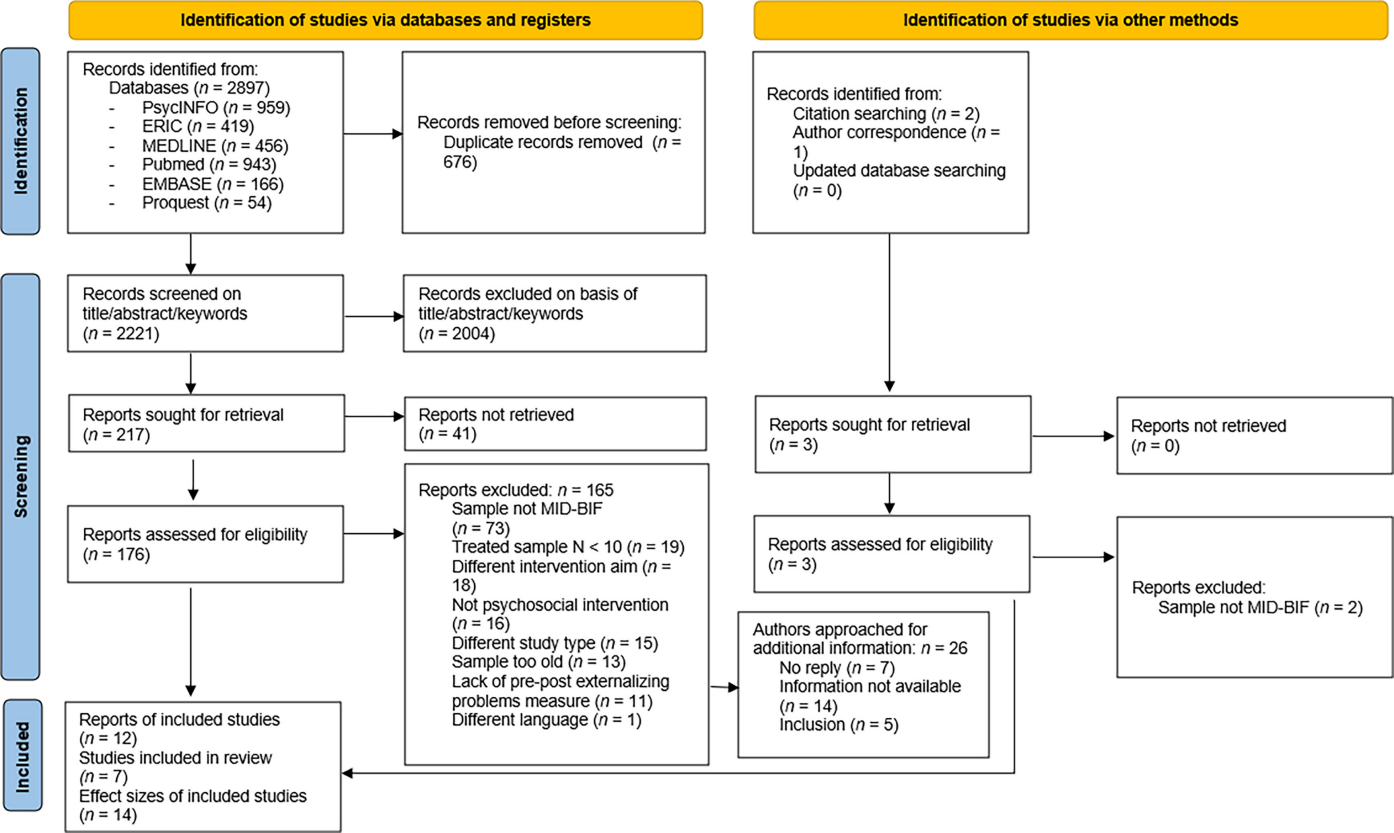
PRISMA flow diagram of the search and selection process.

Figure 1 presents a flowchart of the study selection procedure. In the title‐abstract screening, 33.3% of the papers were double‐screened. In the full‐text screening and coding phases, all papers were independently double‐screened and ‐coded by three authors of this paper (EK, JML, IMK). Any disagreements were discussed and resolved. Interrater reliability in the different screening phases was high (Guildford's *G*s for skewed data > 0.84). Because of the low number of included studies, we were not able to calculate coding interrater reliability (Bujang and Baharum 2017). However, any disagreements were discussed among the pairs of coders and with a third coder if consensus could not be reached. See Supporting Information S3 for a more detailed description of the selection and coding procedures.

#### 2.2.3 Outcome: Externalising Problems

Any continuous outcome measure appropriate for effect size calculation of externalizing problems was used, including any reporter or subdimensions of externalising behaviour (e.g., rule‐breaking or aggressive behaviour). We used post‐ and follow‐up measures of the outcome. All studies provided data on a continuous scale. The rating scales used for each study are shown in Table 2.

#### 2.2.4 Study Characteristics

To include study characteristics in the moderator analyses, we required that at least 70% of the studies provided information on the moderator variable.

##### 2.2.4.1 Participant Characteristics

We coded the number of participants in the analysis. We coded the mean age in years, percentage of girls, and mean IQ. We used categories to code the severity of disability (MID = mean IQ 70 or below, BIF = mean IQ 71 or higher). This variable overlapped with IQ, but more studies reported on MID‐BIF classifications than on specific IQ information.

##### 2.2.4.2 Intervention Characteristics

We coded the number of sessions, session duration in minutes and total intervention length in weeks. We used categories to code intervention format (individual or group format), intervention target (child, parent or combined) and whether an intervention was tailored to the needs of individuals with MID‐BIF in at least one area described by the AAIDD model (Buntinx and Schalock 2010; coded as yes or no). We also coded which AAIDD model areas were addressed in the intervention tailoring.

##### 2.2.4.3 Study Characteristics

We used categories to code measurement method (questionnaire or observation and interview), informant (youth, parent, teacher or clinical staff report), study design (controlled trials or pre‐post‐test design), statistical analyses (intention‐to‐treat or completers only), measurement of clinical significance (i.e., the extent to which an intervention makes a noticeable difference in daily life; Kazdin 1999; coded as yes or no) and measurement of social validity (i.e., the acceptability of treatment's goals, procedures and effects to its consumers; Rapoff 2010; coded as yes or no).

#### 2.2.5 Risk of Bias

We used the Cochrane Collaboration tool for assessing risk of bias (RoB‐2; Sterne et al. 2019), rating the randomisation process, missing outcome data, measurement of the outcome and selection of the reported results. We classified studies into categories of high risk of bias, some concerns or low risk of bias, following the RoB‐2. Two study authors independently double‐coded the studies with the RoB‐2 and discussed any disagreements. Interrater reliability could not be calculated due to the low number of included studies (Bujang and Baharum 2017).

### 2.3 Effect Size Calculation

We calculated two sets of effect sizes for two separate meta‐analyses. As studies with a control group provide more reliable estimates of effectiveness than those without a control group (Wilson and Lipsey 2001), for the first meta‐analysis, we calculated Cohen's *d* as the standard mean change from pre‐test to post‐test between the control and intervention groups. We used the following formula described by Morris (2008): ((*M*_pretreatment_ − *M*_posttreatment_)/SD_pretreatment_) − ((*M*_precontrol_ − *M*_post control_)/SD_precontrol_).

The second meta‐analysis optimised the power for our moderator analysis by including all studies. We calculated Cohen's *d* as the standard mean differences between pre‐test and post‐test of participants receiving the intervention, using the following formula: (*M*_pre_ − *M*_post_)/SD_pre_. We used the standard deviation at pre‐test as a denominator, as there is reason to assume that the interventions influence not only means but also the standard deviations (Lakens 2013). By doing this for all studies, we ensured the type of effect sizes for moderation analyses were comparable across the studies. Our meta‐analyses included studies with small sample sizes (i.e., *n* < 50), and so we applied the following correction to all effect sizes (Card 2012): Hedges' *g* = *d* − (3*d)/(4(*n*_1_ + *n*_2_) − 9). These effect sizes are interpreted similarly to the unadjusted Cohen's *d*. We calculated separate effect sizes for follow‐up data, if available.

### 2.4 Data Synthesis

#### 2.4.1 Data Preparations

We created separate files for controlled trial effect sizes and intervention pre‐post effect sizes using IBM SPSS Version 28.0.1.1. We dummy‐coded categorical moderators and centred all continuous variables around their mean. We checked for outliers (i.e., effect sizes both > 2 SD from the mean and Cook's distance > 4/*N* = 0.285). The study by Acosta et al. (2019) was identified as an outlier among the pre‐test to post‐test effect sizes (*g* = 2.05, Cook's distance = 0.460). We conducted sensitivity analyses without this outlier.

#### 2.4.2 Multilevel Framework

We analysed our data with a multilevel modelling approach using the Metafor Package in R, version 4.3.0 (Viechtbauer 2010). If studies contained multiple measures of externalising problems, this approach allowed us to include all of these in our analyses as separate effect sizes, statistically accounting for the dependency between them (Van den Noortgate et al. 2013). We used a three‐level random‐effects model, estimating the sampling variance for each effect size (Level 1), the variance between effect sizes within studies (Level 2) and the variance in effect sizes between studies (Level 3; Assink and Wibbelink 2016). We used Knapp and Hartung's (2003) method to compute standard errors, which does not assume normality of data (Assink and Wibbelink 2016) and used restricted maximum likelihood (REML; Assink and Wibbelink 2016) to estimate model parameters.

##### 2.4.2.1 Testing Overall Effectiveness

Prior to the analyses, we tested whether there was sufficient power to detect any effects. There is currently no standard approach to determine statistical power for multilevel meta‐analyses (Assink and Wibbelink 2016). Accordingly, we used metapower in R (Griffin 2021) to perform multiple power analyses for regular meta‐analyses, one based on the number of included studies (*n* = 7; i.e., a conservative estimate) and another based on the number of effect sizes (*k* = 14; i.e., a liberal estimate). For both analyses, we found that we had sufficient power to detect intervention effects (power = 0.80–0.98 to detect an effect of 0.40, an average of 60 participants per study and a heterogeneity estimate of 0.50). Thus, for both effect sizes, with and without taking into account the control group, we first tested if the overall effect size of externalising problems was significantly different from 0 by running an intercept‐only model. We created forest plots ordered by the size of the effect estimate to visualise these results.

##### 2.4.2.2 Moderator Analyses

Power analyses indicated that we had insufficient power to detect heterogeneity of effects (power of 0.37–0.76) and moderating effects of variables with two categories (power of 0.11–0.18). As detecting potential moderators of intervention effects was one of the preregistered aims of our meta‐analysis, we conducted moderation analyses for completeness but report on these in Supporting Information S4 (the analysis plan for the moderator analyses can also be found in S4).

#### 2.4.3 Publication Bias

We explored potential publication bias by visually examining a funnel plot. When there is no publication bias, this plot should resemble a symmetrical inverted funnel (Sterne and Egger 2001). We also statistically tested for funnel asymmetry, using Egger's regression test (Egger et al. 1997).

## 3 Results

A total of 12 papers were eligible for our meta‐analysis. Five of these were papers that used duplicate datasets. We thus selected a final sample of seven unique papers with the most comprehensive information for coding. We provide the references to the included and excluded (after full‐text screening) studies, as well as rationale for inclusion and exclusion in Supporting Information S5 and S6.

### 3.1 Participant, Intervention and Study Characteristics

Table 1 presents participant and intervention characteristics for the included studies. The studies were published between 2013 and 2023, although the study by Acosta et al. (2019) used older datasets (Bagner et al. 2010; Bagner and Eyberg 2007). Studies were conducted in India, Ireland, the Netherlands and the United States.

**TABLE 1:** Overview of participant and intervention characteristics of the included studies.

Study	Country	*N*	% Girls	Age *M* (SD)	Age range	IQ *M* (SD)	IQ range	Average ID severity	Intervention type	Intervention context	Number of sessions	Session duration	Intervention length	Tailoring to MID‐BIF
(Acosta et al. 2019)	United States	30	30.3	3.7 (1.05)	1.6–5.3	70.1 (7.32)	56–80	MID	Individual Parent training Aimed at parent	Outpatient	12	75	12 weeks	Did not report tailoring
(Blankestein et al. 2019)	The Netherlands	63 55	39.0	15.0 (1.53)		74.6 (6.99)		BIF	Individual Multisystemic therapy Aimed at family	Home			17.4 weeks	Tailored
(te Brinke et al. 2022)	The Netherlands	42	50	15.5 (1.43)	12–18	75.7 (7.4)	60–84	BIF	Individual CBT Aimed at child	Residential	11	60	14 weeks	Tailored
(Hand et al. 2013)	Ireland	29			6–12				Group Parent training Aimed at parent	School	8	150	8 weeks	Tailored
(Lakhan 2014)	India	26	45.2	10.1 (4.04)	3–13	64.4 (3.98)	55–69	MID	Individual Behavioural modification Aimed at parent and child	Outpatient and home				Did not report tailoring
(McMahon et al. 2023)	Ireland	96	31	14.0 (2.49)	12–19			MID	Group Parent training Aimed at parent	Outpatient	7	135	7 weeks	Tailored
(Schuiringa et al. 2017)	The Netherlands	135	28.4	12.5 (1.99)	9–16	74.2 (10.4)	55–85	BIF	Group Parent training and CBT Aimed at parent and child	Outpatient and day care	22 (sum of parent and child sessions)	82.5	12 weeks	Tailored

Abbreviations: BIF = borderline intellectual functioning; MID = mild intellectual disability.

A total of *N* = 421 participants were included in the studies. Studies included more boys than girls, and most studies included samples with a mean age of at least 10 years. The number of studies with an average ID severity in the MID and the BIF range was similar (three vs. four studies, respectively). One study did not report on ID severity (Hand et al. 2013) but indicated that the participants were recruited from services for children with MID.

Studies primarily examined parent training interventions, as well as one cognitive behavioural therapy intervention, one behaviour modification intervention and one multisystemic therapy intervention. Most interventions took place in an outpatient context, but some also took place in home, special education school, residential or daycare contexts. Both individual and group formats were used to deliver the interventions. Most interventions targeted either parents or had a combined target (parents and children or the entire family). Only one intervention targeted exclusively the child. There was much variation in the number of intervention sessions, session duration and intervention duration.

When considering tailoring to the AAIDD dimensions, five of the included studies reported that they had tailored the intervention to MID‐BIF needs. Most of these studies (i.e., Blankestein et al. 2019; Hand et al. 2013; Schuiringa et al. 2017; te Brinke et al. 2022) reported tailoring to the AAIDD level of intellectual functioning. This included adjustments such as simplification of language, use of visual aids, simplification of content or psycho‐education for parents. Tailoring to the other four AAIDD dimensions was less commonly reported. Two studies (i.e., Blankestein et al. 2019; McMahon et al. 2023) reported tailoring to the level of participation, including the use of specific techniques to motivate and engage families for treatment, and special attention for parents to connect and communicate with their child with MID‐BIF. Two studies (i.e., Blankestein et al. 2019; Schuiringa et al. 2017) reported tailoring to the level of the broader context, including fostering active involvement of the broader social network and information sessions for teachers and group leaders. No studies reported tailoring to the level of adaptive functioning or health problems.

Study characteristics are displayed in Table 2. Most included trials were controlled. Of the controlled trials, there was only one nonrandomised controlled trial (Blankestein et al. 2019), which dealt with the lack of randomisation by using propensity scores. Of the studies with control groups, most used a waitlist control condition. Since only two studies reported effects at follow‐up (Blankestein et al. 2019; McMahon et al. 2023), we did not perform a separate meta‐analysis of follow‐up data. Two of the included studies reported on clinical significance, based on reliable change indices (Acosta et al. 2019, as reported in Bagner and Eyberg 2007) and percentages of children in the (sub)clinical range at pre‐test compared with post‐test (Schuiringa et al. 2017). Social validity of the intervention was addressed in one of the studies, using the Therapy Attitude Inventory (Acosta et al. 2019, as reported in Bagner and Eyberg 2007).

**TABLE 2:** Overview of study characteristics of the included studies.

Study	Study design	Type of analysis	Measure	Method	Clinical significance measure	Social validity measure
(Acosta et al. 2019)	RCT Waitlist control	Intention‐to‐treat	CBCL: Externalising	Parent questionnaire	Yes	Yes
(Blankestein et al. 2019)	CT Active control uCT	Completers only	CBCL: Externalising CBCL: Rulebreaking	Parent questionnaire	No	No
(te Brinke et al. 2022)	uCT	Intention‐to‐treat	YSR: Externalising CBCL: Externalising	Self‐report questionnaire Clinical staff questionnaire	No	No
(Hand et al. 2013)	RCT Waitlist control	Completers only	SDQ: Conduct problems	Parent questionnaire	No	No
(Lakhan 2014)	uCT	Completers only	BASIC‐MR: Violent and destructive Misbehaves with others Rebellious behaviour Antisocial behaviour	Observation and parent interview	No	No
(McMahon et al. 2023)	RCT Waitlist control	Completers only	CAPES‐DD: behavioural problems	Parent questionnaire	No	No
(Schuiringa et al. 2017)	RCT Care as usual control	Intention‐to‐treat	CBCL: Externalising PDR: Externalising TRF: Externalising	Parent questionnaire Parent questionnaire Teacher questionnaire	Yes	No

Abbreviations: BASIC‐MR = Behavioural Assessment Scale for Indian Children with Mental Retardation, CAPES‐DD = Child Adjustment and Parent Efficacy Scale‐Developmental Disability, CBCL = ASEBA Child Behaviour Checklist, CT = (nonrandomised) controlled trial, PDR = Parent Daily Report, RCT = randomised controlled trial, SDQ = Strengths and Difficulties Questionnaire, TRF = ASEBA Teacher Report Form, uCT = uncontrolled trial (pre‐post‐test design), YSR = ASEBA Youth Self‐Report.

### 3.2 Risk of Bias

The risk of bias classification is shown in Supporting Information S7. Of the seven studies included, five showed a high risk of bias and two showed some concerns. The most common cause of high risk of bias was the randomisation process (e.g., studies had no control group, did not use full randomisation or did not conceal condition allocation from researchers). The next most common causes of high risk of bias were deviations from the intended interventions (e.g., completers‐only analysis) and missing outcome data (e.g., no information on how missing data were handled). There was also a high risk of bias because of the outcome measure: All studies relied on subjective measures (e.g., self‐ or parent‐report), rather than more objective measures such as (blinded) observation. However, two studies used multiple informants, with at least one informant not being primarily involved in the intervention (e.g., teachers or clinical staff), thus sufficiently reducing measurement bias for these studies. Lastly, the potential for selective reporting of findings resulted in some concerns for most studies: Only one study explicitly reported that it used prespecified methods and analyses in its study.

### 3.3 Overall Effectiveness

#### 3.3.1 Controlled Trial Effects

**FIGURE 2:**
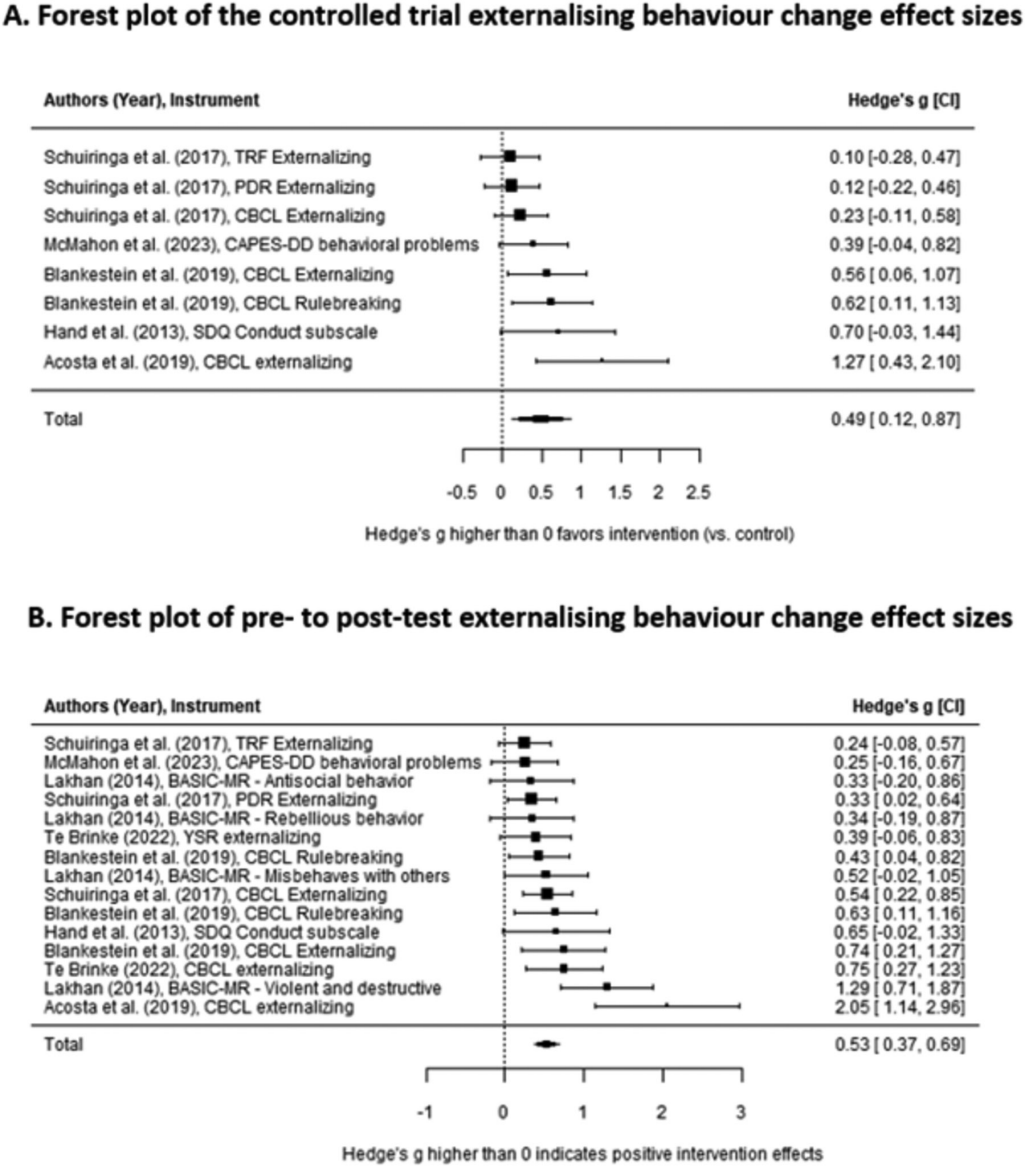
Forest plots of externalising behaviour effect sizes.

The overall effectiveness of the psychosocial interventions on externalising problems, expressed as the standard mean change from pre‐test to post‐test between the control and intervention group, was significant, *g* = 0.49, SE = 0.16, 95% CI [0.12, 0.87], *p* = 0.017. Thus, psychosocial interventions are generally effective at reducing externalising problems of youth with MID‐BIF compared with a control group. A forest plot displaying the effect sizes found in each study is presented in Figure 2A.

#### 3.3.2 Pre‐Test to Post‐Test Effects

The overall effectiveness of the psychosocial interventions on externalising problems, expressed as the standard mean change from pre‐test to post‐test, was significant, *g* = 0.53, SE = 0.08, 95% CI [0.35, 0.70], *p* < 0.001. Thus, youth with MID‐BIF generally show a reduction in externalising problems from pre‐test to post‐test. A forest plot displaying the effect sizes found in each study is presented in Figure 2B.

### 3.4 Moderator Analyses

Although we conducted moderator analyses for completeness, given our low statistical power for these analyses, we only briefly report on the main findings here (the full results are reported in Supporting Information S4). To summarise, we initially found substantial heterogeneity among the pre‐test to post‐test effect sizes, but this was no longer present after the removal of the outlier. Unsurprisingly, we found no evidence that intervention effects depended upon characteristics of participants, intervention or study design.

### 3.5 Publication Bias

**FIGURE 3:**
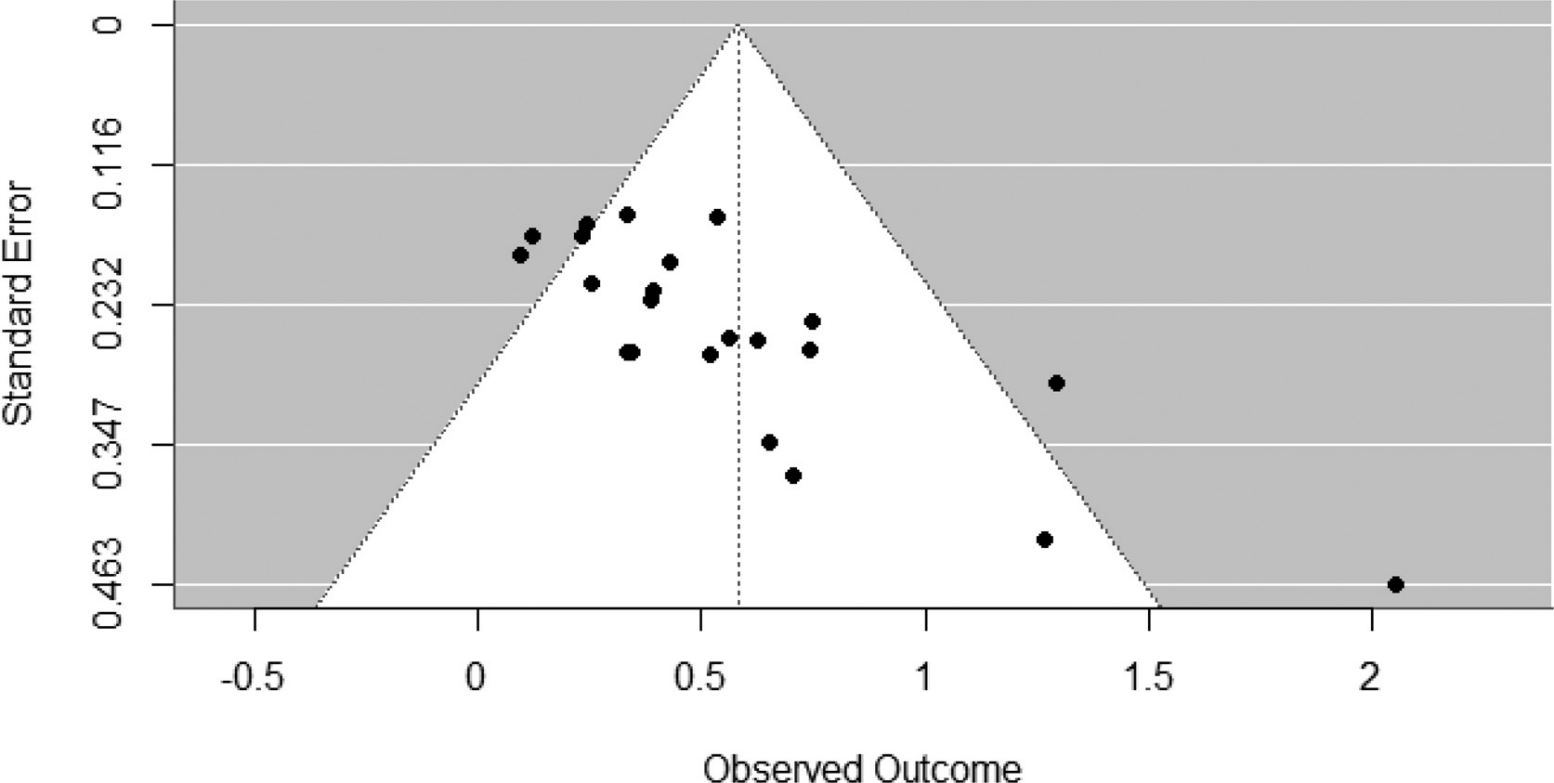
Funnel plot with standard errors along the Y‐axis plotted against effect controlled and pre‐ to post‐test sizes (Hedges' *g*) along the X‐axis.

We explored publication bias using a funnel plot with standard errors plotted against controlled trials and pre‐ to post‐test effect sizes. Visual inspection suggests that the funnel plot is somewhat asymmetrical. Studies with smaller samples (i.e., studies with larger standard errors) appear to be more likely to report larger positive effects, and studies with larger samples (i.e., studies with small standard errors) appear to be more likely to report smaller positive effects (see Figure 3). Egger's regression test confirmed that there was significant funnel plot asymmetry (*Z* = 4.84, *p* < 0.001).

### 3.6 Sensitivity Analyses

When the outlier was excluded, sensitivity analyses showed similar, though slightly smaller effects for both controlled trial effects (*g* = 0.38, SE = 0.14, 95% CI [0.05, 0.71], *p* = 0.030) and pre‐test to post‐test effects (*g* = 0.46, SE = 0.06, 95% CI [0.33, 0.60], *p* < 0.001).

Excluding the outlier did not affect our conclusions about publication bias. There was still significant funnel plot asymmetry (*Z* = 2.91, *p* = 0.004). The funnel plot also looked similar (see Supporting Information S8), suggesting that the overall effect of psychosocial interventions on youth with MID‐BIF's externalising problems is partially driven by smaller studies with relatively strong effects.

## 4 Discussion

In this multilevel meta‐analysis, we investigated the effectiveness of psychosocial interventions targeting externalising problems for youth with MID‐BIF aged up to 18 years old (sample mean age). We identified five controlled and two uncontrolled trials that were eligible, yielding a total of 14 different effect sizes. Our results show that psychosocial interventions for youth with MID‐BIF and externalising problems are generally effective at reducing externalising problems.

Our findings reflect two different types of effect sizes, one for controlled trials (*g* = 0.49) and one for pre‐test to post‐test studies (across all studies; *g* = 0.53), which were similar in magnitude. Although the effect size of controlled trials is assumed to provide a more reliable estimate of intervention effects (Wilson and Lipsey 2001), the pre‐test to post‐test effect size was based on more studies and is likely a more representative reflection of the field. All effect sizes indicated decreased externalising problems (i.e., we found no negative effects). To further interpret the effect sizes we found, we compared them to effect sizes from previous meta‐analyses on externalising problem interventions for youth *without* MID‐BIF as a benchmark (Funder and Ozer 2019). These meta‐analyses have found a mean effect size of 0.42 (range, 0.12–0.98; median effect size, 0.33; Baumel et al. 2021; Boldrini et al. 2023; Granski et al. 2020; Grove et al. 2008; Menting et al. 2013; Riise et al. 2021; Wilson and Lipsey 2007). Thus, the effects of interventions for youth *with* MID‐BIF and externalising problems are similar to those typically found for youth *without* MID‐BIF. Our results are also in line with previous meta‐analyses that investigated the effectiveness of psychosocial interventions for children with a broader range of intellectual disabilities, or a broader range of externalising problems (i.e., psychiatric disorders and challenging behaviour), which had effect sizes ranging between 0.44 and 1.12 (Harvey et al. 2009; Kok et al. 2016).

We did not find heterogeneity in study effect sizes. It is possible that this lack of heterogeneity is (in part) due to common factors in interventions (e.g., the therapeutic alliance and expectancy effects; Cuijpers et al. 2019), leading to similar effects across different interventions. However, given that many previous meta‐analyses on similar externalising problem interventions for youth without MID‐BIF did identify substantial heterogeneity in effects, as well as moderators that explained heterogeneity (e.g., Baumel et al. 2021; Granski et al. 2020; Menting et al. 2013; Riise et al. 2021), it seems more likely that we found no heterogeneity in study effect sizes because of limited statistical power. As the field of interventions for youth with MID‐BIF moves forward, future meta‐analyses may revisit this issue.

### 4.1 Tailoring to MID‐BIF Needs

Most studies reported how they tailored the interventions to the needs of youth with MID‐BIF, in line with at least one of the five areas described in the AAIDD model (Buntinx and Schalock 2010). Most tailoring served to address the difficulties in intellectual functioning. Although this is important (i.e., difficulties in intellectual functioning can theoretically impact an individual's ability to benefit from an intervention; Hronis et al. 2017), intellectual functioning is only one area of life in which individuals with MID‐BIF commonly experience difficulties. Tailoring to two other areas was reported on in some of the studies: tailoring to the level of participation and tailoring to the broader social context. No tailoring in other areas was described. Although we based our conclusions about intervention tailoring on information described in the study, and we thus cannot rule out that some (unreported) tailoring took place, this does suggest that most attention is likely given to tailoring to intellectual functioning, as opposed to the other areas of functioning. In future efforts to tailor interventions to the needs of individuals with MID‐BIF, the different areas of functioning should be more explicitly addressed. Moreover, future publications could benefit from explicitly describing whether and in which areas tailoring took place.

### 4.2 Study Risk of Bias

Another main finding was that most studies included in our meta‐analysis showed a high risk of bias. Previous meta‐analyses have repeatedly identified a generally high risk of bias in intervention studies in the field of intellectual disability intervention research. Kok et al. (2016) identified at least some risk of bias in all included studies in their meta‐analysis of psychosocial interventions for children with MID‐BIF and psychiatric problems. In a meta‐analysis of psychosocial interventions for adults with intellectual disabilities, Graser et al. (2022) also concluded that the risk of bias was generally medium to high. This is concerning, given that it has the potential to inflate effect sizes (Wood et al. 2008). Thus, although our results show a tendency towards the effectiveness of psychosocial interventions for youth with MID‐BIF at reducing externalising problems, we need to be careful in drawing strong conclusions, given the high risk of bias in the included studies.

### 4.3 How Can the Field Move Forward?

In this meta‐analysis, we screened over 2000 unique reports, using a broad search string with the aim to identify as many potentially eligible studies as possible. This resulted in seven unique studies eligible for inclusion. Notably, almost all of these studies were conducted in the last 10 years. This illustrates that the field of intervention studies for youth with MID‐BIF and externalising problems is on the rise.

The relatively high risk of bias in most studies still poses a major challenge to the field. Addressing this challenge is important, as it would give more credibility to the findings concerning intervention effectiveness, potentially empowering clinicians to treat youth with MID‐BIF and externalising problems. To address this challenge, adequately controlled RCTs are needed (cf. Kok et al. 2016). Several studies cited constraints in the clinical context as restricting design options, most commonly leading to problems with randomisation (e.g., the use of incomplete randomisation or inadequate allocation concealment). Some of these challenges can be countered by statistical techniques such as the use of propensity scores (e.g., Blankestein et al. 2019). Moreover, some studies were able to use full randomisation (Acosta et al. 2019; Schuiringa et al. 2017), highlighting that this is also possible in interventions for youth with MID‐BIF in clinical settings.

Other feasible ways to move the field forward and reduce the risk of bias are for studies to include more objective measures of the outcome variables. Although some subjectivity will usually be present in studies into psychosocial interventions, as they often rely on (self‐report) questionnaires, more objectivity can be introduced by applying a multi‐informant approach (e.g., see te Brinke et al. 2022) and the use of observers and/or interviewers (e.g., see Lakhan 2014), which should be blinded when possible. Further, preregistration of analyses can reduce the risk of selective reporting. Only one of the included studies appeared to have followed preregistered analyses (te Brinke et al. 2022). Other ways to reduce the risk of bias in the data‐analyses domain are to use intention‐to‐treat analysis (rather than completers only) and more appropriate handling of missing data, such as using statistical methods that correct for bias (e.g., weighting of participants and likelihood‐based methods), data imputation or the use of sensitivity analyses (Higgins et al. 2019). Lastly, although not related to the risk of bias directly, the reporting on important study information such as age, IQ, gender and diagnoses could be improved in future studies. This would allow for more accurate identification of relevant studies and more accurate moderator analyses in future meta‐analyses.

Our review of the literature identified a number of gaps. Firstly, none of the included intervention studies addressed the broader context of adversity, such as outreach services, parental support or health services (e.g., the Sure Start program; Melhuish et al. 2010). This is important, given that externalising problems are more common in environments of adversity (Metcalf 2016), and youth with MID‐BIF are at increased risk to come from such environments (Emerson et al. 2011). An important goal for future research is to move beyond studying psychological interventions only and to also evaluate social interventions addressing the broader context in which youth with MID‐BIF are growing up. Secondly, only two of the included studies looked beyond intervention effectiveness in terms of statistical significance. A more meaningful evaluation of interventions could be obtained by reporting on the clinical significance of obtained effects and social validity of an intervention (Rapoff 2010). Finally, we identified only one study from a non‐Western country (i.e., India; Lakhan 2014). This is concerning, given that the prevalence, attitudes towards and healthcare for individuals with intellectual disabilities vary widely across cultures (Allison and Strydom 2009). We thus need to build an evidence base that is more globally representative to draw generalisable conclusions about the effectiveness of psychosocial interventions to reduce externalising problems among youth with MID‐BIF.

### 4.4 Strengths and Limitations

This meta‐analysis is the first to investigate the effects of psychosocial interventions for youth with MID‐BIF and externalising problems. We used a multilevel approach that allows for using all available information of individual intervention effects, thus maximising statistical power (Van den Noortgate et al. 2013).

Nonetheless, our meta‐analysis also has a number of limitations. Firstly, there are downsides to including uncontrolled trials, which are more likely to show inflated effect sizes (Wilson and Lipsey 2001). However, as pre‐test to post‐test effect sizes were similar to controlled trial effect sizes, the influence of the inclusion of uncontrolled trials was likely minimal. Secondly, aside from searching dissertations through ProQuest, we did not use alternative approaches to identifying grey literature. We decided against this as it would have introduced a risk of additional selection bias (Ferguson and Brannick 2012), but we cannot be sure that we found all eligible studies from the grey literature. Thirdly, our decision to limit eligibility to English‐language studies may have resulted in missing relevant studies published in other languages (Wood et al. 2008).

Lastly, we excluded studies incorporating single‐case experimental designs (SCED), potentially underrepresenting research fields where such designs are common. For instance, Matson and colleagues (2005) found that most aggression treatment studies for children with intellectual disabilities used applied behaviour analysis evaluated with SCEDs. Although we found no evidence of this trend for youth with MID‐BIF specifically, we may have missed relevant applied behaviour analysis literature. Future meta‐analyses should include SCED studies to build a more comprehensive evidence base (What Works Clearinghouse 2022). Additionally, descriptive research on interventions used for youth with MID‐BIF and externalising problems in practice could clarify gaps between research and real‐world application.

### 4.5 Conclusion

In sum, our results suggest a decrease in externalising problems among youth with MID‐BIF following psychosocial interventions. The magnitude of the effect is similar to effect sizes typically found in intervention research in youth without MID‐BIF. Although this conclusion should be drawn with some caution, because of the still modest number of studies that have been conducted and their risk of bias, these findings are promising. They suggest that psychosocial interventions have the potential to reduce externalising problems among youth with MID‐BIF, thus hopefully encouraging clinicians to implement such interventions in practice. Future studies could incorporate feasible methodological improvements to reduce the risk of bias and comprehensively describe how interventions were tailored to the needs of individuals with MID‐BIF. Nonetheless, the present meta‐analysis provides initial evidence for the effectiveness of psychosocial interventions for reducing externalising problems in youth with MID‐BIF and highlights the need for methodological improvements to move the field forward.

## Ethics Statement

This study was exempt from ethical review within the Ethics Review Board of the Faculty of Social & Behavioural Sciences at Utrecht University, as it concerns a meta‐analysis using only aggregate data that cannot be tracked back to individuals.

## Conflicts of Interest

The authors declare no conflicts of interest.

## Supporting information


**Data S1.** Elaborated PICOS selection criteria.
**Data S2.** Literature final search strings.
**Data S3.** Elaborate description of selection and coding procedures.
**Data S4.**Analyses plan and results of the moderator analyses.
**Data S5.** References to included studies.
**Data S6.** References to excluded studies.
**Data S7.** Table: Risk of bias evaluation.
**Data S8.** Figure: Sensitivity analysis funnel plot with standard errors along the Y‐Axis plotted against effect controlled and pre‐ to post‐test sizes (Hedge's g) along the X‐Axis, after removal of the outlier.
**Data S9.** PRISMA checklist.

## Data Availability

Our study protocol, data and analysis code are available on Open Science Framework (https://doi.org/10.17605/OSF.IO/9RTX7).
