# Supplementary material for: Effectiveness of Psychosocial Interventions for Youth With Mild Intellectual Disabilities or Borderline Intellectual Functioning and Externalising Problems: A Multilevel Meta‐Analysis
Source: J Intellect Disabil Res. 2025 Jul 14;69(9):795–808. doi: 10.1111/jir.70014 (PMC12418733; doi:10.1111/jir.70014)
Supplement: Supplementary file 1 — Data S1. Elaborated PICOS selection criteria. Data S2. Literature final search strings. Data S3. Elaborate description of selection and coding procedures. Data S4.Analyses plan and results of the moderator analyses. Data S5. References to included studies. Data S6. References to excluded studies. Data S7. Table: Risk of bias evaluation. Data S8. Figure: Sensitivity analysis funnel plot with standard errors along the Y‐Axis plotted against effect controlled and pre‐ to post‐test sizes (Hedge's g) along the X‐Axis, after removal of the outlier. Data S9. PRISMA checklist. [file JIR-69-795-s001.docx]

**Appendix A:** Supplement to Kühl, E., Koning, I. M., Deković, M., Thomaes, S., & Liber, J. M.

[S1. Elaborated PICOS selection criteria 2](#_Toc193207396)

[S2. Literature final search strings 5](#_Toc193207397)

[S3. Elaborate description of selection and coding procedures 9](#_Toc193207398)

[S4. Analyses plan and results of the moderator analyses 11](#_Toc193207399)

[S5. References to included studies 15](#_Toc193207400)

[S6. References to excluded studies 17](#_Toc193207401)

[S7. Table: Risk of bias evaluation 29](#_Toc193207402)

[S8. Figure: Sensitivity analysis funnel plot with standard errors along the Y-Axis plotted against effect controlled and pre- to post-test sizes (Hedge’s g) along the X-Axis, after removal of the outlier 30](#_Toc193207403)

[S9. PRISMA checklist 31](#_Toc193207404)

# S1. Elaborated PICOS selection criteria

***Population***

We included youth with MID-BIF (IQ between 55-85, or a specific mention of MID-BIF diagnosis), with a mean age ≤ 18 years old and individual participants’ age ≤ 23 years old. The mean age criterion of 18 was chosen, because a transition from paediatric to adult services occurs around 18 years old in many countries (e.g., the Netherlands, the United Kingdom; Andersson Elffers Felix, 2024; National Health services, 2024). We chose a higher cut-off for the individual participant’s age, because we expected to encounter some mixed-age samples, due to pronounced differences in developmental and chronological age among youth with MID-BIF (De Wit et al., 2012). We chose the cut-off of 23 years old, as to not immediately exclude these mixed-age samples, but also not extent our samples too much into the early young adulthood population. Studies were included if they reported specifically on a (sub)sample of children with MID-BIF. If a sample consisted of participants with MID-BIF as well as other participants (i.e., with lower or higher intellectual functioning), authors were contacted and asked if they could provide results for the MID-BIF subsample only. If authors did not reply or were unable to provide this information, the study was excluded. We also excluded studies if they sampled participants with severe sensory impairments (hearing, vision) that would affect the implementation of the intervention, or if their intervention condition sample size was smaller than *n* = 10.

***Intervention***

We included studies if they investigated a psychosocial intervention which had as one of the primary aims to reduce externalising problems. The intervention could focus on youth themselves, their parents/caregivers, their teachers, or staff (or a combination of them). The intervention could be implemented in diverse contexts (e.g., community-based settings, residential settings, schools, home, or outpatient clinics). We excluded studies that focused on pharmacological intervention were.

***Outcome***

We included studies with at least one outcome measure of change in youth’s externalising problems (defined as oppositional defiant, rule-breaking, and/or aggressive behaviour, following the symptoms of disruptive, impulse-control, and conduct disorders from the DSM-5; American Psychiatric Association, 2013). Outcomes measuring exclusively hyperactive behaviour we not included, because ADHD is classified as a neurodevelopmental disorder, assumed to have different origin (Metcalf, 2016). We included studies focusing on outcomes of challenging behaviour if they encompassed (subscales about) exclusively externalising problems (e.g., aggressive behaviour).

***Study design and comparison***

We included both controlled (randomized and non-randomized) and non-controlled intervention studies with (at least) one pre- and one post-intervention measure of externalising problems. We excluded studies analysing externalising problems at the individual level (i.e., [multiple] single case). These studies require specific approaches to analyse (e.g., (Heyvaert et al., 2012) and cannot be directly compared to studies with group-comparison, due incomparable effect size calculations (Van den Noortgate & Onghena, 2008). We included studies with all types of control groups (e.g., waitlist, care-as-usual), as well as studies without a control group.

**References**

American Psychiatric Association. (2013). *Diagnostic and statistical manual of mental disorders* (5th ed.). https://doi.org/https://doi.org/10.1176/appi.books.9780890425596

Andersson Elffers Felix. (2024). *De overgang van jeugdhulp naar volwassenheid*. chrome-extension://efaidnbmnnnibpcajpcglclefindmkaj/https://open.overheid.nl/documenten/344497a1-9784-47be-b122-97585eff0452/file

De Wit, M., Moonen, X., & Douma, J. (2012). *Richtlijnen Effectieve Interventies LVB [Guidlines for effective interventions MID]*. Dekkers.

Heyvaert, M., Maes, B., Van den Noortgate, W., Kuppens, S., & Onghena, P. (2012). A multilevel meta-analysis of single-case and small-n research on interventions for reducing challenging behavior in persons with intellectual disabilities. *Research in Developmental Disabilities*, *33*(2), 766–780. https://doi.org/10.1016/j.ridd.2011.10.010

Metcalf, E. B. (2016). ADHD and the disruptive behavior disorders. In *The Medical Basis of Psychiatry: Fourth Edition* (pp. 333–369). Springer New York. https://doi.org/10.1007/978-1-4939-2528-5_18

National Health services. (2024, March 11). *Moving from children’s social care to adults’ social care*. Secretary of State for Health and Social Care. https://www.nhs.uk/conditions/social-care-and-support-guide/caring-for-children-and-young-people/moving-from-childrens-social-care-to-adults-social-care/

Van den Noortgate, W., & Onghena, P. (2008). A multilevel meta-analysis of single-subject experimental design studies. *Evidence-Based Communication Assessment and Intervention*, *2*(3), 142–151. https://doi.org/10.1080/17489530802505362

# S2. Literature final search strings

Database: PsycINFO (Ovid)

Search date August 21^st^ 2023

__________________________________________________________________________________

1. exp intellectual development disorder/ or developmental disabilities/
2. (Intellectual* Disab* or Developmental* Disab* or Borderline Intellectual Functioning or Mental* Retard* or Intellectual* Retard* or Mental* Handicap* or Developmental* Delay* or Cognitive* Impair*).ti,ab,id.
3. exp therapy/ or exp intervention/ or exp psychotherapy/
4. (Psychotherap* or Training* or Therap* or intervention* or prevention or parent* program*).ti,id.
5. Behavior problems/ or exp disruptive behavior disorders/ or behavior disorders/
6. (Externali* or Behavio* Disorder* or Behavio* problem* or Aggress* or Oppositional Defiant Disorder* or Conduct Disorder* or Conduct Problem* or Disruptive behavio*).ti,ab,id.
7. (Child* or Adolescen* or Youth* or P?ediatric).ti,ab,id.
8. 1 or 2
9. 3 or 4
10. 5 or 6
11. 8 and 9 and 10 and 7
12. 11 not (physical treatment methods/ or drug therapy/)
13. limit 12 to (english language and ("0110 peer-reviewed journal" or "0400 dissertation abstract"))

Database: ERIC (Ovid)

Search date August 21^st^ 2023

__________________________________________________________________________________

1. exp Intellectual Disability or developmental delays/ or developmental disabilities/
2. (Intellectual* Disab* or Developmental* Disab* or Borderline Intellectual Functioning or Mental* Retard* or Intellectual* Retard* or Mental* Handicap* or Developmental* Delay* or Cognitive* Impair*).ti,ab,id.
3. exp intervention/ or exp therapy/ or exp program effectiveness/
4. (Psychotherap* or Training* or Therap* or intervention* or prevention or parent* program*).ti,id.
5. behavior problems/ or aggression/ or behavior disorders/ or antisocial behavior/
6. (Externali* or Behavio* Disorder* or Behavio* problem* or Aggress* or Oppositional Defiant Disorder* or Conduct Disorder* or Conduct Problem* or Disruptive behavio*).ti,ab,id.
7. children/ or adolescents/
8. (Child* or Adolescen* or Youth* or P?ediatric).ti,ab,id.
9. 1 or 2
10. 3 or 4
11. 5 or 6
12. 7 or 8
13. 9 and 10 and 11 and 12
14. 13 not exp drug therapy/
15. limit 14 to (english language and (journal articles or "dissertations/theses - doctoral dissertations"))

Database: MEDLINE (Ovid)

Search date August 21^st^ 2023

__________________________________________________________________________________

1. exp intellectual disability/ or developmental disabilities/
2. (Intellectual* Disab* or Developmental* Disab* or Borderline Intellectual Functioning or Mental* Retard* or Intellectual* Retard* or Mental* Handicap* or Developmental* Delay* or Cognitive* Impair*).ti,ab,kf.
3. exp psychotherapy/
4. (Psychotherap* or Training* or Therap* or intervention* or prevention or parent* program*).ti,kf.
5. Problem behavior/ or "Attention Deficit and Disruptive Behavior Disorders"/
6. (Externali* or Behavio* Disorder* or Behavio* problem* or Aggress* or Oppositional Defiant Disorder* or Conduct Disorder* or Conduct Problem* or Disruptive behavio*).ti,ab,kf.
7. (adolescent/ or child/
8. (Child* or Adolescen* or Youth* or Pediatric or Paediatric).ti,ab,kf.
9. 1 or 2
10. 3 or 4
11. 5 or 6
12. 7 or 8
13. 9 and 10 and 11 and 12
14. 13 not exp drug therapy/
15. limit 14 to (english language and journal article)

Database: Pubmed (Pubmed)

Search date August 21^st^ 2023

__________________________________________________________________________________

1. "intellectual disability"[MeSH Terms]
2. ("intellectual disab*"[Title/Abstract] OR "intellectually disab*"[Title/Abstract] OR "developmental disab*"[Title/Abstract] OR "developmentally disab*"[Title/Abstract] OR "borderline intellectual functioning"[Title/Abstract] OR "mental retard*"[Title/Abstract] OR "mentally retard*"[Title/Abstract] OR "intellectual retard*"[Title/Abstract] OR "intellectually retard*"[Title/Abstract] OR "mental handicap*"[Title/Abstract] OR "mentally handicap*"[Title/Abstract] OR "developmental delay*"[Title/Abstract] OR "developmentally delay*"[Title/Abstract] OR "cognitive impair*"[Title/Abstract] OR "cognitively impair*"[Title/Abstract])
3. "Psychotherapy"[Mesh]
4. ("psychotherap*"[Title/Abstract] OR "intervention*"[Title/Abstract] OR "training*"[Title/Abstract] OR "therap*"[Title/Abstract])
5. "Problem Behavior"[Mesh]
6. ("externali*"[Title/Abstract] OR "behavior disorder*"[Title/Abstract] OR "behaviour disorder*"[Title/Abstract] OR "behavior problem*"[Title/Abstract] OR "behaviour problem*"[Title/Abstract] OR "aggress*"[Title/Abstract] OR "oppositional defiant disorder*"[Title/Abstract] OR "conduct disorder*"[Title/Abstract] OR "conduct problem*"[Title/Abstract] OR "disruptive behavio*"[Title/Abstract])
7. ("Child"[Mesh] OR "Adolescent"[Mesh])
8. ("child*"[Title/Abstract] OR "adolescen*"[Title/Abstract] OR "youth*"[Title/Abstract] OR "Pediatric"[Title/Abstract] OR "Paediatric"[Title/Abstract])
9. (#1 OR #2) AND (#3 OR #4) AND (#5 OR #6) AND (#7 OR #8)
10. #9 NOT "Drug Therapy"[MeSH Terms]
11. #10 AND "english"[Language]

Database: EMBASE (Embase.com)

Search date August 21^st^ 2023

__________________________________________________________________________________

1. 'mentally disabled person'/exp OR 'intellectual impairment'/de
2. 'intellectual disab*':ti,ab,kw OR 'intellectually disab*':ti,ab,kw OR 'developmental disab*':ti,ab,kw OR 'developmentally disab*':ti,ab,kw OR 'borderline intellectual functioning':ti,ab,kw OR 'mental retard*':ti,ab,kw OR 'mentally retard*':ti,ab,kw OR 'intellectual retard*':ti,ab,kw OR 'intellectually retard*':ti,ab,kw OR 'mental handicap':ti,ab,kw OR 'mentally handicap*':ti,ab,kw OR 'developmental delay*':ti,ab,kw OR 'developmentally delay*':ti,ab,kw OR 'cognitive impair*':ti,ab,kw OR 'cognitively impair*':ti,ab,kw
3. #1 or #2
4. 'psychotherapy'/exp
5. 'psychotherap*':ti,kw OR 'intervention*':ti,kw OR 'training*':ti,kw OR 'therap*':ti,kw OR 'prevention':ti,kw OR 'parent program*':ti,kw OR 'parenting program*':ti,kw
6. #4 or #5
7. 'problem behavior'/de
8. 'externali*' OR 'behavior disorder*' OR 'behaviour disorder*' OR 'behavior problem*' OR 'behaviour problem*' OR 'aggress*' OR 'oppositional defiant disorder*' OR 'conduct disorder*' OR 'conduct problem*' OR 'disruptive behavio*'
9. #7 or #8
10. 'child'/exp OR 'adolescent'/exp
11. 'child*' OR 'adolescen*' OR 'youth*' OR 'pediatric' OR 'paediatric'
12. #10 or #11
13. #3 and #6 and #9 and #12
14. #13 NOT 'drug therapy'/exp)
15. #14 AND [embase]/lim NOT ([embase]/lim AND [medline]/lim)
16. #15 AND ('article'/it OR 'article in press'/it)

Database: Proquest (proquest.com)

Search date August 21^st^ 2023

__________________________________________________________________________________

(noft((Intellectual* Disab*) OR (Developmental* Disab*) OR (borderline intellectual functioning) OR (mental* retard*) OR (intellectual* retard*) OR (mental* handicap*) OR (developmental* delay*) OR (cognitive* impair*)) OR MAINSUBJECT.EXACT("Severe Intellectual Disability") OR MAINSUBJECT.EXACT("Developmental Disabilities") OR MAINSUBJECT.EXACT("Moderate Intellectual Disability") OR MAINSUBJECT.EXACT("Mild Intellectual Disability") OR MAINSUBJECT.EXACT("Down Syndrome") OR MAINSUBJECT.EXACT("Intellectual Disability") OR MAINSUBJECT.EXACT("Developmental Delays"))

AND

(noft(psychotherapy* OR intervention* OR training* OR therap*) OR MAINSUBJECT.EXACT(“Intervention”) OR MAINSUBJECT.EXACT(“Therapy”) OR MAINSUBJECT.EXACT(“Psychoherapy”))

AND

(noft(externali* OR (behavi* disorder*) OR (behavi* problem*) OR aggress* OR (oppositional defiant disorder) OR (conduct disorder*) OR (conduct problem*) OR (challenging behavi*) OR (disruptive behavi*)) OR MAINSUBJECT.EXACT(“Behavior problems”) OR MAINSUBJECT.EXACT(“Aggression”) OR MAINSUBJECT.EXACT(“Behavior disorders”) OR MAINSUBJECT.EXACT(“Antisocial behavior”))

AND

(noft (child* OR adolescen* OR youth* OR pediatric OR paediatric) OR MAINSUBJECT.EXACT("Children") OR MAINSUBJECT.EXACT("Adolescents"))

Limit to:
- Dissertation
- Dissertation/Thesis
- 041: Dissertations/Theses - Doctoral Dissertations
- Doctoral Dissertation
- 040: Dissertations/Theses

# S3. Elaborate description of selection and coding procedures

***Study selection***

First, the first author (E. Kühl) and a research assistant used Rayyan (Ouzzani et al., 2016) to screen title, abstract and keywords for potentially relevant papers. We double screened a third (33.3%) of the articles, yielding a high inter-rater reliability of Guildford’s *G* .84 (this reliability index best takes into account skewed data; Silveira & Siqueira, 2022). Second, three authors of this paper (E. Kühl, J. M. Liber, and I. Koning) independently double-screened the full-text in pairs of two, yielding a high interrater-reliability of Guildford’s *G* .86. The three screeners discussed and resolved any disagreements. If the full text was unavailable or if quantitative information necessary for effect size computation was not provided in the paper, we requested this information from the authors if contact information was available. Some studies included not exclusively participants with MID-BIF (e.g., also including more severe disabilities) or the severity of the disabilities were unclear. In these cases (*n* = 23), we also contacted authors to request effect size information of participants specifically in the MID-BIF range, as we only included the studies of which the MID-BIF sample information could be extracted. Six authors did not reply, 14 were unable to provide the requested information, and 5 papers were included after receiving additional information.

After study selection, the reference lists from the selected articles were screened by the first author to ensure no relevant articles were missed. This was done stepwise as well: 1) screening of titles, 2) screening of abstracts, 3) screening of full papers. This yielded one new study for inclusion.

**Coding**

Three authors of this study independently double-coded all studies (either E. Kühl and J. M. Liber, or E. Kühl and I. Koning). Due to the low number of studies, we were not able to calculate coding interrater reliability (Bujang & Baharum, 2017). Therefore, any disagreements were discussed among the pairs of coders. If consensus could not be reached, the third coder involved in the coding process was consulted. If any outcome or moderator data was missing, we approach study authors to retrieve these. All outcome data were retrieved, but not all authors were able to provide missing moderator data. In these cases, listwise deletion was used.

**References**

Bujang, M. A., & Baharum, N. (2017). Guidelines of the minimum sample size requirements for Cohen’s Kappa. *Epidemiology Biostatistics and Public Health*, *14*(2), e12267-1-e12267-10. https://doi.org/10.2427/12267

Ouzzani, M., Hammady, H., Fedorowicz, Z., & Elmagarmid, A. (2016). Rayyan-a web and mobile app for systematic reviews. *Systematic Reviews*, *5*(1). https://doi.org/10.1186/s13643-016-0384-4

Silveira, P. S. P., & Siqueira, J. O. (2022). Better to be in agreement than in bad company: A critical analysis of many kappa-like tests. *Behavior Research Methods*, *55*, 3326–3347. https://doi.org/10.3758/s13428-022-01950-0

# S4. Analyses plan and results of the moderator analyses

**Analyses plan for the moderator analyses**

Power analyses indicated that we had insufficient power to detect heterogeneity of effects (power of .37 – .76) and moderating effects of variables with two categories (power of .11 – .18). As detecting potential moderators of intervention effects was one of the preregistered aims of our meta-analyses, we conducted moderation analyses as planned. While still reporting significance test for completeness, we decided not to rely on these given the low power, and instead report on effect size differences descriptively. Accordingly, our discussion of moderation effects should be interpreted with due caution.

We first tested for the effect sizes without the control group (pretest to posttest changes), if there was significant heterogeneity within studies (level 2) and between studies (level 3), by conducting two one-sided log-likelihood ratio tests from the intercept-only model. We divided the *p*-values from the R output by two, as R by default performs two-sided tests (Assink & Wibbelink, 2016). We examined how the total variance was distributed over the three levels. If the log-likelihood ratio tests were not significant, we still considered heterogeneity sufficient to proceed with moderation analyses if < 75% of the total amount of variance was located at level 1 (i.e., 75% rule; Hunter & Schmidt, 1990). In case of sufficient heterogeneity, we conducted moderating analyses, by extending the intercept-only model with a mixed-effects multilevel model. We evaluated moderation effects in separate models, to avoid the inflation of type II error rates due to multicollinearity (Hox et al., 2018). Interpretations of significance should therefore follow Holm-Bonferroni correction for multiple testing (α = 0.00625; Holm, 1979).

**Results of the moderator analyses**

Neither the loglikelihood ratio test at the within-study variance level (*χ^2^*(1) = 0.641, *p* = .212), nor at the between-study variance level (*χ^2^*(1) = 0.00, *p* = .500) was significant. Most of the total variance could be attributed to variance at level 1 (i.e., sampling variance; 60.9%) and the rest could be attributed to variance at level 2 (i.e., within-study variance; 39.1%). None of the total variance could be attributed to level 3 (i.e., between-study variance; 0.0%). Thus, there was substantial heterogeneity among the pretest to posttest effect sizes, according to the 75% rule (Hunter & Schmidt, 1990).

Possibly due to low power, none of the intervention, participant, or study design characteristics significantly moderated intervention effectiveness (see Table S1). Considering the effect sizes descriptively, we noted that none of the regression coefficients for the continuous moderators were large enough to be potentially meaningful. Concerning categorical intervention characteristics, we observed that effect sizes tended to be larger for individual interventions, compared to group-based interventions. Effects of interventions targeting parents tended to be larger, compared to interventions targeting youth or a combined approach that targeted both parents and youth. Effects for interventions that reported no tailoring tended to be slightly larger compared to those that did. Concerning participant characteristics, we observed slightly larger effects for studies with a MID sample, compared to a BIF sample. Concerning design characteristics, we observed some differences across informants, with the largest effect sizes for effects based on staff-report and parent-report, and smaller effects sizes for effects based on youth self-report or teacher-report. Studies with high bias tended to have larger effects, compared to studies with some concerns.

We conducted sensitivity analyses without the identified outlier. Heterogeneity was largely driven by the outlier. With the outlier removed, the 75% rule no longer applied: almost all variance was located at level 1 (95%) and the remaining variance was located at level 2 (5%). Log-likelihood ratio tests remained non-significant. Subsequently, looking at moderator effects was no longer justified.

**References**

Assink, M., & Wibbelink, C. J. M. (2016). Fitting three-level meta-analytic models in R: A step-by-step

tutorial. *The Quantitative Methods for Psychology*, *12*(3), 154–174. <https://doi.org/10.20982/tqmp.12.3.p154>

Holm, S. (1979). A simple sequentially rejective multiple test procedure. *Scandinavian Journal of*

*Statistics*, *6*(2), 65–70. <http://www.jstor.org/stable/4615733>

Hox, J., Moerbeek, M., & Van de Schoot, R. (2018). *Multilevel Analysis: Techniques and Applications*.

Routledge.

Hunter, J. E., & Schmidt, F. L. (1990). *Methods of meta-analysis : Correcting error and bias in research*

*findings*. Sage.

| **Table S1**  *Moderating effects for externalising behaviour pretest to posttest change outlier included* | | | | | | | | | | | |
| --- | --- | --- | --- | --- | --- | --- | --- | --- | --- | --- | --- |
| **Potential continuous moderators** | | | | | | | | | | | |
| **Intervention characteristics** | | | | | ***k*** | ***n*** | ***β*** | **95%*CI*** | ***SE*** | ***F* (df1, df2)**^a^ | ***p***^b^ |
| Number of sessions | | | | | 8 | 5 | -.01 | -0.15, 0.14 | .06 | 0.01 (1, 6) | .912 |
| Session duration | | | | | 8 | 5 | -.01 | -0.03, 0.02 | .01 | 0.35 (1, 6) | .576 |
| Intervention length | | | | | 10 | 6 | .02 | -0.12, 0.15 | .06 | 0.06 (1, 8) | .808 |
| **Participant characteristics** | | | | |  |  |  |  |  |  |  |
| Mean age | | | | | 13 | 6 | -.09 | -0.18, 0.00 | .04 | 4.96 (1, 11) | .048 |
| Percentage girls | | | | | 13 | 6 | -.01 | -0.06, 0.04 | .02 | 0.17 (1, 11) | .684 |
| Mean IQ | | | | | 12 | 5 | -.02 | -0.15, 0.11 | .06 | 0.15 (1, 10) | .712 |
| **Design characteristics** | | | | |  |  |  |  |  |  |  |
| Number of participants | | | | | 15 | 7 | < -.01 | -0.01, 0.00 | < .01 | 3.62 (1, 12) | .081 |
| **Potential categorical moderators** | | | | | | | | | | | |
| **Intervention characteristics** | | | | | ***k*** | ***n*** | ***g*** | **95%*CI*** | ***SE*** | ***F* (df1, df2)**^a^ | ***p***^b^ |
| Intervention format | | | | |  |  |  |  |  | 3.35 (1, 12) | .092 |
|  | | | Individual (RC) | | 9 | 4 | 0.63*** | 0.42, 0.85 | 0.10 |  |  |
|  | | | Group | | 5 | 3 | 0.37** | 0.15, 0.60 | 0.10 |  |  |
| Intervention target | | | | |  |  |  |  |  | 0.34 (2, 11) | .719 |
|  | | | Self (RC) | | 2 | 1 | 0.56 | -0.38, 1.50 | 0.43 |  |  |
|  | | | Parent | | 3 | 3 | 0.81* | 0.15, 1.49 | 0.30 |  |  |
|  | | | Combined | | 9 | 3 | 0.50 | -0.02, 1.03 | 0.24 |  |  |
| **Participant characteristics** | | | | |  |  |  |  |  |  |  |
| Severity of disability | | | | |  |  |  |  |  | 0.91 (1, 11) | .361 |
|  | | MID (RC) | | | 6 | 3 | 0.64** | 0.32, 0.97 | 0.15 |  |  |
|  | | BIF | | | 7 | 3 | 0.47** | 0.21, 0.72 | 0.12 |  |  |
| **Design characteristics** | | | | |  |  |  |  |  |  |  |
| Measurement method | | | | |  |  |  |  |  | 0.11 (1, 12) | .745 |
|  | Questionnaire (RC) | | | | 10 | 6 | 0.53*** | 0.29, 0.76 | 0.11 |  |  |
|  | Interview/observation | | | | 4 | 1 | 0.60* | 0.17, 1.03 | 0.20 |  |  |
| Informant | | | | |  |  |  |  |  | 0.56 (3, 10) | .654 |
|  | Child/adolescent (RC) | | | | 1 | 1 | 0.39 | -0.36, 1.14 | 0.34 |  |  |
|  | Parent | | | | 11 | 6 | 0.58*** | 0.34, 0.81 | 0.11 |  |  |
|  | Teacher | | | | 1 | 1 | 0.24 | -0.42, 0.91 | 0.30 |  |  |
|  | Staff | | | | 1 | 1 | 0.75 | -0.03, 1.52 | 0.35 |  |  |
| Study design | | | | |  |  |  |  |  | 0.08 (1, 12) | .787 |
|  | | | Pre-posttest design (RC) | | 7 | 3 | 0.56*** | 0.29, 0.83 | 0.12 |  |  |
|  | | | Controlled trail | | 7 | 5 | 0.51*** | 0.26, 0.77 | 0.12 |  |  |
| Analysis | | | | |  |  |  |  |  | < 0.01 (1, 8) | .965 |
|  | | | | Completers only (RC) | 1 | 1 | 0.65 | -0.64, 1.95 | 0.56 |  |  |
|  | | | | Intention-to-treat | 9 | 5 | 0.63* | 0.12, 1.13 | 0.22 |  |  |
| Risk of bias | | | | |  |  |  |  |  | 4.10(1, 12) | .066 |
|  | | | | Some concerns | 4 | 2 | 0.35** | 0.13, 0.58 | .10 |  |  |
|  | | | | High bias | 10 | 5 | 0.63*** | 0.43, 0.83 | .09 |  |  |
| *Note*. *k* = number of effect sizes, *n* = number of studies, *β* = regression coefficient based on *t*-distribution, *CI* = confidence interval, *SE* = standard error, *g* = Mean Hedges’ *g* in the reference category based on *t*-distribution.  ^a^ Omnibus test of all regression coefficients in the model.  ^b^ *p-*value of the omnibus test, significant at *p* < .00625.  * *p* < .05  ** *p* < .01.  *** *p* < .001. | | | | | | | | | | | |

# S5. References to included studies

References include reports on duplicate datasets. Articles in bold were selected as the most appropriate reports and therefore used for the coding phase.

**^1^Acosta, J., Garcia, D., & Bagner, D. M. (2019). Parent-child interaction therapy for children with developmental delay: The role of sleep problems. *Journal of Developmental and Behavioral Pediatrics*,*40*(3), 183–191.**

[**https://doi.org/doi:https://dx.doi.org/10.1097/DBP.0000000000000647**](https://doi.org/doi:https://dx.doi.org/10.1097/DBP.0000000000000647)*****

^1^Bagner, D. M. (2013). Father's role in parent training for children with developmental delay. *Journal of Family Psychology*,*27*(4), 650–657.

<https://doi.org/doi:https://dx.doi.org/10.1037/a0033465>*

^1^Bagner, D. M., & Eyberg, S. M. (2007). Parent-child interaction therapy for disruptive behavior in children with mental retardation: A randomized controlled trial. *Journal of Clinical Child and Adolescent Psychology*,*36*(3), 418–429.

<https://doi.org/doi:https://dx.doi.org/10.1080/15374410701448448>*

^1^Bagner, D. M., & Graziano, P. A. (2013). Barriers to success in parent training for young children with developmental delay: The role of cumulative risk. *Behavior Modification*,*37*(3), 356–377.

<https://doi.org/doi:https://dx.doi.org/10.1177/0145445512465307>*

**Blankestein, A., Rijken, R., Eeren, H. V., Lange, A., Scholte, R., Moonen, X., Vuyst, K. D., Leunissen, J., & Didden, R. (2019). Evaluating the effects of multisystemic therapy for adolescents with intellectual disabilities and antisocial or delinquent behaviour and their parents. *Journal of Applied Research in Intellectual Disabilities*,*32*(3), 575–590.**

[**https://doi.org/10.1111/jar.12551**](https://doi.org/10.1111/jar.12551)******

**Brinke, L. W. t., Schuiringa, H. D., Menting, A. T. A., Dekovic, M., Westera, J. J., & Castro, B. O. d. (2022). Treatment approach and sequence effects in cognitive behavioral therapy targeting emotion regulation among adolescents with externalizing problems and intellectual disabilities. *Cognitive Therapy and Research*,*46*(2), 302–318.**

[**https://doi.org/doi:https://dx.doi.org/10.1007/s10608-021-10261-1**](https://doi.org/doi:https://dx.doi.org/10.1007/s10608-021-10261-1)

**Hand, A., Raghallaigh, C. N., Cuppage, J., Coyle, S., & Sharry, J. (2013). A controlled clinical evaluation of the Parents Plus Children's Programme for parents of children aged 6-12 with mild intellectual disability in a school setting. *Clinical Child Psychology and Psychiatry*,*18*(4), 536–555.**[**https://doi.org/doi:https://dx.doi.org/10.1177/1359104512460861**](https://doi.org/doi:https:/dx.doi.org/10.1177/1359104512460861)

^2^Herwaarden, A. v., Schuiringa, H., Nieuwenhuijzen, M. v., Castro, B. O. d., Lochman, J. E., & Matthys, W. (2022). Therapist alliance building behavior and treatment adherence for Dutch children with mild intellectual disability or borderline intellectual functioning and externalizing problem behavior. *Research in developmental disabilities*,*128*, 104296.

<https://doi.org/doi:https://dx.doi.org/10.1016/j.ridd.2022.104296>

^1^Kimonis, E. R., Bagner, D. M., Linares, D., Blake, C. A., & Rodriguez, G. (2014). Parent training outcomes among young children with callous-unemotional conduct problems with or at risk for developmental delay. *Journal of Child and Family Studies*,*23*(2), 437–448.

<https://doi.org/doi:https://dx.doi.org/10.1007/s10826-013-9756-8>*

**Lakhan, R. (2014). Behavioral management in children with intellectual disabilities in a resource-poor setting in Barwani, India. *Indian Journal of Psychiatry*,*56*(1), 39–45.**

[**https://doi.org/doi:10.4103/0019-5545.124712**](https://doi.org/doi:10.4103/0019-5545.124712)*****

**McMahon, S. M., Wilson, C., and Sharry, J. . (2023). Parents Plus parenting programme for parents of adolescents with intellectual disabilities: A cluster randomised controlled trial. *Journal of Applied Research in Intellectual Disabilities*,*36*(4), 871–880.**

[**https://doi.org/10.1111/jar.13105**](https://doi.org/10.1111/jar.13105)*****

**^2^Schuiringa, H., Nieuwenhuijzen, M. v., Castro, B. O. d., Lochman, J. E., & Matthys, W. (2017). Effectiveness of an intervention for children with externalizing behavior and mild to borderline intellectual disabilities: A randomized trial. *Cognitive Therapy and Research*,*41*(2), 237–251.**[**https://doi.org/doi:https://dx.doi.org/10.1007/s10608-016-9815-8**](https://doi.org/doi:https:/dx.doi.org/10.1007/s10608-016-9815-8)******

^1^ These studies were published on (parts of) the same dataset. The study by Acosta et al. (2019) was ultimately selected for the coding, as it most comprehensively reported on the MID-BIF sample. The other studies reported on only a part of the MID-BIF sample (Bagner et al., 2007; Kimonis et al., 2014) or did not report on pretest to posttest outcomes of externalising behaviour for the full sample (Bagner, 2013; Bagner et al., 2013).

^2^ Studies with duplicate datasets. The study by Schuiringa et al. (2017) was ultimately selected for the coding, as it most comprehensively reported on the effectiveness of the intervention.

* Study originally reported on a sample with not exclusively MID-BIF participants, so effect size information and sample characteristics were requested for specifically this part of the sample.

** Full sample was appropriate, but additional information was requested to for calculation of effect sizes.

# S6. References to excluded studies

Studies could be excluded for multiple reasons. However, they are listed here under the reason highest up in our screening hierarchy (i.e., the same order as listed below).

**Different study type**

Management of behaviour problems in children with mental handicap. (1987). *Lancet*,*1*(8532), 545-546.

Blasi, V., Baglio, G., Baglio, F., Canevini, M. P., & Zanette, M. (2017). Movement cognition and narration of the emotions treatment versus standard speech therapy in the treatment of children with borderline intellectual functioning: A randomized controlled trial. *BMC Psychiatry*,*17*. [https://doi.org/https://dx.doi.org/10.1186/s12888-017-1309-z](https://doi.org/https:/dx.doi.org/10.1186/s12888-017-1309-z)

Coren, E., Hutchfield, J., Thomae, M., & Gustafsson, C. (2010). Parent training support for intellectually disabled parents. *The Cochrane database of systematic reviews*(6), CD007987. [https://doi.org/https://dx.doi.org/10.1002/14651858.CD007987.pub2](https://doi.org/https:/dx.doi.org/10.1002/14651858.CD007987.pub2)

Court, D., & Harris, M. (1965). Child care in general practice. Speech disorders in children. I. *British medical journal*,*2*(5457), 345-347.

Crnic, K. A., Neece, C. L., McIntyre, L. L., Blacher, J., & Baker, B. L. (2017). Intellectual disability and developmental risk: Promoting intervention to improve child and family well-being. *Child Development*,*88*(2), 436-445. [https://doi.org/https://dx.doi.org/10.1111/cdev.12740](https://doi.org/https:/dx.doi.org/10.1111/cdev.12740)

Fuller, J. L., & Fitter, E. A. (2020). Mindful parenting: A behavioral tool for parent well-being. *Behavior Analysis in Practice*,*13*(4), 767-771. [https://doi.org/https://dx.doi.org/10.1007/s40617-020-00447-6](https://doi.org/https:/dx.doi.org/10.1007/s40617-020-00447-6)

Goldstein, E. A., & Eisenberg, L. (1965). Review of psychiatric progress 1964. Child psychiatry; mental deficiency. *The American journal of psychiatry*,*121*, 655-659.

Hartelius, H. (1965). A study of male juvenile delinquents. *Acta psychiatrica Scandinavica. Supplementum*,*182*.

Krakowski, A. J. (1963). The role of the physician in the management of the emotionally disturbed child. IV Management. *Psychosomatics*,*4*, 270-278.

Laugeson, E. A., Paley, B., Schonfeld, A. M., Carpenter, E. M., Frankel, F., & O'Connor, M. J. (2007). Adaptation of the children's friendship training program for children with fetal alcohol spectrum disorders. *Child & Family Behavior Therapy*,*29*(3), 57-69.

Leffler, J. M., Vaughn, A. J., & Thompson, A. D. (2021). Acute, intensive, and residential services (airs) for youth: Introduction to special issue. *Evidence-Based Practice in Child and Adolescent Mental Health*,*6*(4), 421-423. <https://doi.org/10.1080/23794925.2021.1996301>

McDiarmid, M. D., & Bagner, D. M. (2005). Parent child interaction therapy for children with disruptive behavior and developmental disabilities. *Education and Treatment of Children*,*28*(2), 130-141.

Pelc, K., Kornreich, C., Foisy, M. L., & Dan, B. (2006). Recognition of emotional facial expressions in attention-deficit hyperactivity disorder. *Pediatr Neurol*,*35*(2), 93-97. <https://doi.org/10.1016/j.pediatrneurol.2006.01.014>

Philbrick, W., Reynolds, S. L., & Ross, R. (1965). Special education for the mentally, physically and emotionally handicapped: summary of a state program. *Clinical pediatrics*,*4*, 404-408.

West, S. C., & Kaniok, P. (2009). Strategies for crisis intervention and prevention-revised as a current proposal in care of individuals with intellectual disabilities and challenging behaviours. *International Journal of Special Education*,*24*(1), 1-7.

**Size of treated sample below *N* < 10**

Ackerman, K. B., Spriggs, A. D., & Rhodes, A. L. (2021). Peer mediators use of prompting to increase social communication in students with disabilities. *Communication Disorders Quarterly*,*43*(1), 42-50. <https://doi.org/10.1177/1525740120936999>

Acotto, C. (2015). The iPad as an alternative reinforcer during Functional Communication Training: Effects on self-injury and aggression. *Dissertation Abstracts International Section A: Humanities and Social Sciences*,*76*(1).

Aldosari, M. S. (2017). Efficacy of choice of preferred engagement stimuli on escape-maintained disruptive behavior. *International Journal of Special Education*,*32*(3), 472-484.

Brophy, A. A. (2011). Effects of a social skill instruction program on the social skill acquisition of african american high school students with mild intellectual disabilities and challenging behaviors. *ProQuest LLC*.

Carr, E. G., & Carlson, J. I. (1993). Reduction of severe behavior problems in the community using a multicomponent treatment approach. *Journal of Applied Behavior Analysis*,*26*(2), 157-172.

Denkowski, G. C., & Denkowski, K. M. (1985). Community-based residential treatment of the mentally retarded adolescent offender: Phase 1, reduction of aggression behavior. *Journal of Community Psychology*,*13*(3), 299-305. [https://doi.org/https://dx.doi.org/10.1002/1520-6629%28198507%2913:3%3C299::AID-JCOP2290130308%3E3.0.CO;2-5](https://doi.org/https:/dx.doi.org/10.1002/1520-6629%28198507%2913:3%3C299::AID-JCOP2290130308%3E3.0.CO;2-5)

Douglas, A. (2021). The use of behavior skills training and SAFMEDS to teach routine-based behavior support plans to parents using telehealth in the home setting. *Dissertation Abstracts International Section A: Humanities and Social Sciences*,*82*(10).

Embregts, P. J. C. M. (2003). Using self-management, video feedback, and graphic feedback to improve social behavior of youth with mild mental retardation. *Education and Training in Developmental Disabilities*,*38*(3), 283-295.

Fisher, W. W., Greer, B. D., Querim, A. C., & DeRosa, N. (2014). Decreasing excessive functional communication responses while treating destructive behavior using response restriction. *Research in developmental disabilities*,*35*(11), 2614-2623. [https://doi.org/https://dx.doi.org/10.1016/j.ridd.2014.06.024](https://doi.org/https:/dx.doi.org/10.1016/j.ridd.2014.06.024)

Hagopian, L. P., Kuhn, S. A. C., Long, E. S., & Rush, K. S. (2005). Schedule thinning following communication training: using competing stimuli to enhance tolerance to decrements in reinforcer density. *Journal of Applied Behavior Analysis*,*38*(2), 177-193.

Hall, H. V., Price, A. B., Shinedling, M., Peizer, S. B., & Massey, R. H. (1973). Control of aggressive behavior in a group of retardates using positive and negative reinforcement procedures. *The Training school bulletin*,*70*(3), 179-186.

Kahng, S. W., Abt, K. A., & Schonbachler, H. E. (2001). Assessment and treatment of low-rate high-intensity problem behavior. *Journal of Applied Behavior Analysis*,*34*(2), 225-228.

Kellner, M. H., & Tutin, J. (1995). A school-based anger management program for developmentally and emotionally disabled high school students. *Adolescence*,*30*(120), 813-825.

LaBrot, Z. C., Kupzyk, S., Strong-Bak, W., Pasqua, J. L., & Mahon, J. (2020). Examination of group-based behavioral skills training for parents of children with intellectual and neurodevelopmental disorders. *Child & Family Behavior Therapy*,*42*(2), 98-124. [https://doi.org/https://dx.doi.org/10.1080/07317107.2020.1738715](https://doi.org/https:/dx.doi.org/10.1080/07317107.2020.1738715)

Lodewijks, H. P. B. (2011). Treatment of moderately intellectually disabled delinquent youth in a dutch juvenile justice facility with closed and open units. *Learning and Individual Differences*,*21*(1), 49-54.

Luiselli, J. K., & Slocumb, P. R. (1983). Management of multiple aggressive behaviors by differential reinforcement. *Journal of behavior therapy and experimental psychiatry*,*14*(4), 343-347.

McComas, J. J., Thompson, A., & Johnson, L. (2003). The effects of presession attention on problem behavior maintained by different reinforcers. *Journal of Applied Behavior Analysis*,*36*(3), 297-307. [https://doi.org/https://dx.doi.org/10.1901/jaba.2003.36-297](https://doi.org/https:/dx.doi.org/10.1901/jaba.2003.36-297)

Morrey, J. G. (1971). Parent training in precise behavior management with mentally retarded children. *Dissertation Abstracts International Section A: Humanities and Social Sciences*,*31*(7), 3376.

Singh, N. N., Chan, J., Karazsia, B. T., McPherson, C. L., & Jackman, M. M. (2017). Tele-health training of teachers to teach a mindfulness-based procedure for selfmanagement of aggressive behavior to students with intellectual and developmental disabilities. *International Journal of Developmental Disabilities*,*63*(4), 195-203. <https://doi.org/10.1080/20473869.2016.1277841>

**Sample not MID-BIF**

Bagner, D. M., Berkovits, M. D., Coxe, S., Frech, N., Garcia, D., Golik, A., Heflin, B. H., Heymann, P., Javadi, N., Sanchez, A. L., Wilson, M. K., & Comer, J. S. (2023). Telehealth treatment of behavior problems in young children with developmental delay: A randomized clinical trial. *JAMA Pediatr*,*177*(3). <https://doi.org/10.1001/jamapediatrics.2022.5204>

Brightman, R. P., Baker, B. L., Clark, D. B., & Ambrose, S. A. (1982). Effectiveness of alternative parent training formats. *Journal of behavior therapy and experimental psychiatry*,*13*(2), 113-117. [https://doi.org/https://dx.doi.org/10.1016/0005-7916%2882%2990051-9](https://doi.org/https:/dx.doi.org/10.1016/0005-7916%2882%2990051-9)

Campbell, C. (2012). Adapting an evidence-based intervention to improve social and behavioral competence in Head Start children: Evaluating the effectiveness of teacher-child interaction training. *Theses, Dissertations, and Student Research: Department of Psychology*, 37.

Carr, E. G., & Blakeley-Smith, A. (2006). Classroom Intervention for Illness-Related Problem Behavior in Children With Developmental Disabilities. *Behavior Modification*,*30*(6), 901-924. [https://doi.org/https://dx.doi.org/10.1177/0145445506290080](https://doi.org/https:/dx.doi.org/10.1177/0145445506290080)

Carrasco, J. M. (2011). The impact of treatment intensity on a parent and child therapy program. *Dissertation Abstracts International: Section B: The Sciences and Engineering*,*71*(10), 6433.

Coughlin, M., Sharry, J., Fitzpatrick, C., Guerin, S., & Drumm, M. (2009). A controlled clinical evaluation of the parents plus children's programme: A video-based programme for parents of children aged 6 to 11 with behavioural and developmental problems. *Clinical Child Psychology and Psychiatry*,*14*(4), 541-558. [https://doi.org/https://dx.doi.org/10.1177/1359104509339081](https://doi.org/https:/dx.doi.org/10.1177/1359104509339081)

Davidson, B. C., Davis, E., Cadenas, H., Barnett, M., Sanchez, B. E. L., Gonzalez, J. C., & Jent, J. (2021). Universal teacher-child interaction training in early special education: A pilot cluster-randomized control trial. *Behavior Therapy*,*52*(2), 379-393. [https://doi.org/https://dx.doi.org/10.1016/j.beth.2020.04.014](https://doi.org/https:/dx.doi.org/10.1016/j.beth.2020.04.014)

Dur, V. M., & Carr, E. G. (1992). An analysis of maintenance following functional communication training. *Journal of Applied Behavior Analysis*,*25*(4), 777-794. [https://doi.org/https://dx.doi.org/10.1901/jaba.1992.25-777](https://doi.org/https:/dx.doi.org/10.1901/jaba.1992.25-777)

Dur, V. M., Hieneman, M., Clarke, S., Wang, M., & Rinaldi, M. L. (2013). Positive family intervention for severe challenging behavior I: A multisite randomized clinical trial. *Journal of Positive Behavior Interventions*,*15*(3), 133-143. [https://doi.org/https://dx.doi.org/10.1177/1098300712458324](https://doi.org/https:/dx.doi.org/10.1177/1098300712458324)

Esbensen, A. J., Hoffman, E. K., Beebe, D. W., Byars, K., Carle, A. C., Epstein, J. N., & Johnson, C. (2022). Randomized behavioral sleep clinical trial to improve outcomes in children with down syndrome. *American Journal on Intellectual and Developmental Disabilities*,*127*(2), 149-164.

Feldman, M. A., Condillac, R. A., Tough, S., Hunt, S., & Griffiths, D. (2002). Effectiveness of community positive behavioral intervention for persons with developmental disabilities and severe behavior disorders. *Behavior Therapy*,*33*(3), 377-398. [https://doi.org/https://dx.doi.org/10.1016/S0005-7894%2802%2980034-X](https://doi.org/https:/dx.doi.org/10.1016/S0005-7894%2802%2980034-X)

Glazemakers, I., & Deboutte, D. (2013). Modifying the 'positive parenting program' for parents with intellectual disabilities. *Journal of Intellectual Disability Research*,*57*(7), 616-626. [https://doi.org/https://dx.doi.org/10.1111/j.1365-2788.2012.01566.x](https://doi.org/https:/dx.doi.org/10.1111/j.1365-2788.2012.01566.x)

Gore, N., & Umizawa, H. (2011). Challenging behavior training for teaching staff and family carers of children with intellectual disabilities: A preliminary evaluation. *Journal of Policy and Practice in Intellectual Disabilities*,*8*(4), 266-275. [https://doi.org/https://dx.doi.org/10.1111/j.1741-1130.2011.00315.x](https://doi.org/https:/dx.doi.org/10.1111/j.1741-1130.2011.00315.x)

Griffin, C., Guerin, S., Sharry, J., & Drumm, M. (2010). A multicentre controlled study of an early intervention parenting programme for young children with behavioural and developmental difficulties. *International Journal of Clinical and Health Psychology*,*10*(2), 279-294.

Groves, E. A., & Austin, J. L. (2019). Does the Good Behavior Game evoke negative peer pressure? Analyses in primary and secondary classrooms. *Journal of Applied Behavior Analysis*,*52*(1), 3-16. [https://doi.org/https://dx.doi.org/10.1002/jaba.513](https://doi.org/https:/dx.doi.org/10.1002/jaba.513)

Hetzroni, O. E. (2003). A positive behaviour support: A preliminary evaluation of a school-wide plan for implementing AAC in a school for students with intellectual disabilities. *Journal of Intellectual and Developmental Disability*,*28*(3), 283-296. [https://doi.org/https://dx.doi.org/10.1080/1366825031000150955](https://doi.org/https:/dx.doi.org/10.1080/1366825031000150955)

Holtz, C. A., Carrasco, J. M., Mattek, R. J., & Fox, R. A. (2009). Behavior problems in toddlers with and without developmental delays: Comparison of treatment outcomes. *Child & Family Behavior Therapy*,*31*(4), 292-311. [https://doi.org/https://dx.doi.org/10.1080/07317100903311018](https://doi.org/https:/dx.doi.org/10.1080/07317100903311018)

Hoogsteder, L. M., Stams, G.-J. J. M., Schippers, E. E., & Bonnes, D. (2018). Responsive Aggression Regulation Therapy (Re-ART): An evaluation study in a Dutch juvenile justice institution in terms of recidivism. *International Journal of Offender Therapy and Comparative Criminology*,*62*(14), 4403-4424. [https://doi.org/https://dx.doi.org/10.1177/0306624X18761267](https://doi.org/https:/dx.doi.org/10.1177/0306624X18761267)

Houck, G. M., & King, M. C. (1993). Cognitive functioning, and behavioral and emotional adjustment in maltreated children post-intervention. *Journal of child and adolescent psychiatric and mental health nursing*,*6*(2), 5-17.

Hudson, A., Cameron, C., & Matthews, J. (2008). The wide-scale implementation of a support program for parents of children with an intellectual disability and difficult behaviour. *Journal of Intellectual and Developmental Disability*,*33*(2), 117-126. [https://doi.org/https://dx.doi.org/10.1080/13668250802065885](https://doi.org/https:/dx.doi.org/10.1080/13668250802065885)

Hudson, A. M., Matthews, J. M., Gavidia-Payne, S. T., Cameron, C. A., Mildon, R. L., Radler, G. A., & Nankervis, K. L. (2003). Evaluation of an intervention system for parents of children with intellectual disability and challenging behaviour. *Special Issue on Family Research.*,*47*(4), 238-249. [https://doi.org/https://dx.doi.org/10.1046/j.1365-2788.2003.00486.x](https://doi.org/https:/dx.doi.org/10.1046/j.1365-2788.2003.00486.x)

Inoue, M., Kishimoto, T., & Fukuzaki, T. (2021). Interventions for students with problem behaviors: A workshop incorporating applied behavior analysis for japanese teachers. *Yonago acta medica*,*64*(1), 98-106. [https://doi.org/https://dx.doi.org/10.33160/yam.2021.02.013](https://doi.org/https:/dx.doi.org/10.33160/yam.2021.02.013)

J. V. Fechter, J. (1971). Modeling and environmental generalization by mentally retarded subjects of televised aggressive or friendly behavior. *American journal of mental deficiency*,*76*(2), 266-267.

Kanoy, K. W., & Schroeder, C. S. (1985). Suggestions to parents about common behavior problems in a pediatric primary care office: Five years of follow-up. *Journal of Pediatric Psychology*,*10*(1), 15-30. [https://doi.org/https://dx.doi.org/10.1093/jpepsy/10.1.15](https://doi.org/https:/dx.doi.org/10.1093/jpepsy/10.1.15)

Kong, M. M.-y., & Au, T. K.-f. (2018). The incredible years parent program for Chinese preschoolers with developmental disabilities. *Early Education and Development*,*29*(4), 494-514. [https://doi.org/https://dx.doi.org/10.1080/10409289.2018.1461987](https://doi.org/https:/dx.doi.org/10.1080/10409289.2018.1461987)

Kostulski, M., Breuer, D., & Dopfner, M. (2021). Does parent management training reduce behavioural and emotional problems in children with intellectual disability? A randomised controlled trial. *Research in developmental disabilities*,*114*, 103958. [https://doi.org/https://dx.doi.org/10.1016/j.ridd.2021.103958](https://doi.org/https:/dx.doi.org/10.1016/j.ridd.2021.103958)

Lardén, M., Högström, J., & Långström, N. (2021). Effectiveness of an individual cognitive-behavioral intervention for serious, young male violent offenders: Randomized controlled study with twenty-four-month follow-up. *Frontiers in Psychiatry*,*12*. <https://doi.org/10.3389/fpsyt.2021.670957>

Lee, Y., Keown, L. J., S, M. R., & ers. (2022). The effectiveness of the Stepping Stones Triple P seminars for Korean families of a child with a developmental disability. *Heliyon*,*8*(6), e09686. [https://doi.org/https://dx.doi.org/10.1016/j.heliyon.2022.e09686](https://doi.org/https:/dx.doi.org/10.1016/j.heliyon.2022.e09686)

Leung, C., Chan, S., Lam, T., Yau, S., & Tsang, S. (2016). The effect of parent education program for preschool children with developmental disabilities: A randomized controlled trial. *Research in developmental disabilities*,*56*, 18-28. [https://doi.org/https://dx.doi.org/10.1016/j.ridd.2016.05.015](https://doi.org/https:/dx.doi.org/10.1016/j.ridd.2016.05.015)

Leung, C., Fan, A., S, M. R., & ers. (2013). The effectiveness of a Group Triple P with Chinese parents who have a child with developmental disabilities: A randomized controlled trial. *Research in developmental disabilities*,*34*(3), 976-984. [https://doi.org/https://dx.doi.org/10.1016/j.ridd.2012.11.023](https://doi.org/https:/dx.doi.org/10.1016/j.ridd.2012.11.023)

Leung, C., Lai, C., Lau, D., Leung, S., & Pin, T. W. (2020). Effectiveness of a multidisciplinary parent training program for children with developmental disabilities: A single-blind randomized waitlist controlled trial. *Journal of Child Health Care*,*24*(4), 560-576. [https://doi.org/https://dx.doi.org/10.1177/1367493519880447](https://doi.org/https:/dx.doi.org/10.1177/1367493519880447)

Louw, C., & Grobler, H. (2015). The utilisation of the bridging technique during therapy to overcome contact-making barriers in adolescents. *Journal of Social Work Practice*,*29*(2), 173-190. [https://doi.org/https://dx.doi.org/10.1080/02650533.2014.930818](https://doi.org/https:/dx.doi.org/10.1080/02650533.2014.930818)

Matson, J. L., Fee, V. E., Coe, D. A., & Smith, D. (1991). A social skills program for developmentally delayed preschoolers. *Journal of Clinical Child Psychology*,*20*(4), 428-433. [https://doi.org/https://dx.doi.org/10.1207/s15374424jccp2004_11](https://doi.org/https:/dx.doi.org/10.1207/s15374424jccp2004_11)

May, F. S., McLean, L. A., A., erson, Hudson, A., Cameron, C., & Matthews, J. (2013). Father participation with mothers in the Signposts program: An initial investigation. *Journal of Intellectual and Developmental Disability*,*38*(1), 39-47. [https://doi.org/https://dx.doi.org/10.3109/13668250.2012.748184](https://doi.org/https:/dx.doi.org/10.3109/13668250.2012.748184)

McIntyre, L. L. (2008). Adapting Webster-Stratton's incredible years parent training for children with developmental delay: Findings from a treatment group only study. *Journal of Intellectual Disability Research*,*52*(12), 1176-1192. [https://doi.org/https://dx.doi.org/10.1111/j.1365-2788.2008.01108.x](https://doi.org/https:/dx.doi.org/10.1111/j.1365-2788.2008.01108.x)

McIntyre, L. L. (2008). Parent training for young children with developmental disabilities: Randomized controlled trial. *American Journal on Mental Retardation*,*113*(5), 356-368. [https://doi.org/https://dx.doi.org/10.1352/2008.113:356-368](https://doi.org/https:/dx.doi.org/10.1352/2008.113:356-368)

Monlux, K. D., Pollard, J. S., Buj, A. Y., Rodriguez, a., & Hall, S. S. (2022). Conducting In-Home Functional Analyses of Aggression and Self-Injury Exhibited by Boys with Fragile X Syndrome. *Journal of developmental and behavioral pediatrics : JDBP*,*43*(4), e237-e245. [https://doi.org/https://dx.doi.org/10.1097/DBP.0000000000001019](https://doi.org/https:/dx.doi.org/10.1097/DBP.0000000000001019)

Mulligan, B., John, M., Coombes, R., & Singh, R. (2015). Developing outcome measures for a family intensive support service for children presenting with challenging behaviours. *British Journal of Learning Disabilities*,*43*(3), 161-167. [https://doi.org/https://dx.doi.org/10.1111/bld.12091](https://doi.org/https:/dx.doi.org/10.1111/bld.12091)

Orim, M. A., Orim, S. O., Adeleke, P. O., Essien, E. E., Olayi, J. E., Essien, C. K., Dada, O. A., Ewa, J. A., Eke, V. U., Igba, I. U., Ogar, R. O., & Owan, V. J. (2022). Cognitive behavioral therapy as treatment intervention for aggressive behaviors in clients with intellectual disabilities and concomitant mental health conditions. *Journal of education and health promotion*,*11*(1), 395-. [https://doi.org/https://dx.doi.org/10.4103/jehp.jehp_545_22](https://doi.org/https:/dx.doi.org/10.4103/jehp.jehp_545_22)

Page, T. J., Perrin, F. A., Tessing, J. L., Vorndran, C. M., & Edmonds, D. (2007). Beyond treatment of individual behavior problems: An effective residential continuum of care for individuals with severe behavior problems. *Special Issue: The treatment and assessment of the severe behavior of individuals with autism and developmental disabilities.*,*22*(1), 35-45. [https://doi.org/https://dx.doi.org/10.1002/bin.229](https://doi.org/https:/dx.doi.org/10.1002/bin.229)

Paluck, R. J., & Esser, A. H. (1971). Controlled experimental modification of aggressive behavior in territories of severely retarded boys. *American journal of mental deficiency*,*76*(1), 23-29.

Peters-Scheffer, N., Didden, R., Mulders, M., & Korzilius, H. (2010). Low intensity behavioral treatment supplementing preschool services for young children with autism spectrum disorders and severe to mild intellectual disability. *Research in developmental disabilities*,*31*(6), 1678-1684. [https://doi.org/https://dx.doi.org/10.1016/j.ridd.2010.04.008](https://doi.org/https:/dx.doi.org/10.1016/j.ridd.2010.04.008)

Peters-Scheffer, N., Didden, R., Mulders, M., & Korzilius, H. (2013). Effectiveness of low intensity behavioral treatment for children with autism spectrum disorder and intellectual disability. *Research in Autism Spectrum Disorders*,*7*(9), 1012-1025. [https://doi.org/https://dx.doi.org/10.1016/j.rasd.2013.05.001](https://doi.org/https:/dx.doi.org/10.1016/j.rasd.2013.05.001)

Phillips, C. L., Iannaccone, J. A., Rooker, G. W., & Hagopian, L. P. (2017). Noncontingent reinforcement for the treatment of severe problem behavior: An analysis of 27 consecutive applications. *Journal of Applied Behavior Analysis*,*50*(2), 357-376. [https://doi.org/https://dx.doi.org/10.1002/jaba.376](https://doi.org/https:/dx.doi.org/10.1002/jaba.376)

Prieto-Bayard, M., & Baker, B. L. (1986). Parent training for Spanish-speaking families with a retarded child. *Journal of Community Psychology*,*14*(2), 134-143. [https://doi.org/https://dx.doi.org/10.1002/1520-6629%28198604%2914:2%3C134::AID-JCOP2290140204%3E3.0.CO;2-6](https://doi.org/https:/dx.doi.org/10.1002/1520-6629%28198604%2914:2%3C134::AID-JCOP2290140204%3E3.0.CO;2-6)

Quetsch, L. B., Bradley, R. S., Theodorou, L., Newton, K., & McNeil, C. B. (2022). Community-based Agency Delivery of Parent-Child Interaction Therapy: Comparing Outcomes for Children with and Without Autism Spectrum Disorder and/or Developmental Delays. *Journal of Autism and Developmental Disorders*. [https://doi.org/https://dx.doi.org/10.1007/s10803-022-05755-0](https://doi.org/https:/dx.doi.org/10.1007/s10803-022-05755-0)

Quinn, M., Carr, A., Carroll, L., & O'Sullivan, D. (2007). Parents Plus programmes I: Evaluation of its effectiveness for pre-school children with developmental disabilities and behavioural problems. *Journal of Applied Research in Intellectual Disabilities*,*20*(4), 345-359. [https://doi.org/https://dx.doi.org/10.1111/j.1468-3148.2006.00352.x](https://doi.org/https:/dx.doi.org/10.1111/j.1468-3148.2006.00352.x)

Roberts, C., Mazzucchelli, T., Studman, L., S, M. R., & ers. (2006). Behavioral Family Intervention for Children With Developmental Disabilities and Behavioral Problems. *Journal of Clinical Child and Adolescent Psychology*,*35*(2), 180-193. [https://doi.org/https://dx.doi.org/10.1207/s15374424jccp3502_2](https://doi.org/https:/dx.doi.org/10.1207/s15374424jccp3502_2)

Ros, R., Hern, J., ez, Graziano, P. A., & Bagner, D. M. (2016). Parent training for children with or at risk for developmental delay: The role of parental homework completion. *Behavior Therapy*,*47*(1), 1-13. [https://doi.org/https://dx.doi.org/10.1016/j.beth.2015.08.004](https://doi.org/https:/dx.doi.org/10.1016/j.beth.2015.08.004)

Roux, B., & Philippot, P. (2020). A mindfulness-based program among adolescent boys with behavior disorders: A quasi-experimental study. *Journal of Child and Family Studies*,*29*(8), 2186-2200. [https://doi.org/https://dx.doi.org/10.1007/s10826-020-01751-z](https://doi.org/https:/dx.doi.org/10.1007/s10826-020-01751-z)

Roux, G., Sofronoff, K., S, M., & ers. (2013). A randomized controlled trial of group Stepping Stones Triple P: a mixed-disability trial. *Family process*,*52*(3), 411-424. [https://doi.org/https://dx.doi.org/10.1111/famp.12016](https://doi.org/https:/dx.doi.org/10.1111/famp.12016)

Ruane, A., Carr, A., Moffat, V., Finn, T., Murphy, A., O'Brien, O., Groarke, H., & O'Dwyer, R. (2019). A randomised controlled trial of the Group Stepping Stones Triple P training programme for parents of children with developmental disabilities. *Clinical Child Psychology and Psychiatry*,*24*(4), 728-753. [https://doi.org/https://dx.doi.org/10.1177/1359104519827622](https://doi.org/https:/dx.doi.org/10.1177/1359104519827622)

Salisbury, M. R., Roos, L. E., Horn, S. R., Peake, S. J., & Fisher, P. A. (2022). The effectiveness of KEEP for families of children with developmental delays: Integrating FIND video coaching into Parent Management Training--Oregon Model: A randomized trial. *Prevention Science*,*23*(6), 1029-1040.

Schieltz, K. M., Wacker, D. P., Harding, J. W., Berg, W. K., Lee, J. F., Dalmau, Y. C. P., Mews, J., & Ibrahimovic, M. (2011). Indirect effects of functional communication training on non-targeted disruptive behavior. *Special Issue: Evaluating assistive technology in the education of persons with severe disabilities.*,*20*(1), 15-32. [https://doi.org/https://dx.doi.org/10.1007/s10864-011-9119-8](https://doi.org/https:/dx.doi.org/10.1007/s10864-011-9119-8)

Schuurmans, A. A. T., Nijhof, K. S., Engels, R. C. M. E., & Granic, I. (2018). Using a videogame intervention to reduce anxiety and externalizing problems among youths in residential care: An initial randomized controlled trial. *Journal of Psychopathology and Behavioral Assessment*,*40*(2), 344-354. [https://doi.org/https://dx.doi.org/10.1007/s10862-017-9638-2](https://doi.org/https:/dx.doi.org/10.1007/s10862-017-9638-2)

Shapiro, C. J., Kilburn, J., & Hardin, J. W. (2014). Prevention of behavior problems in a selected population: Stepping stones triple P for parents of young children with disabilities. *Research in developmental disabilities*,*35*(11), 2958-2975. [https://doi.org/https://dx.doi.org/10.1016/j.ridd.2014.07.036](https://doi.org/https:/dx.doi.org/10.1016/j.ridd.2014.07.036)

Sharry, J., Guerin, S., Griffin, C., & Drumm, M. (2005). An evaluation of the parents plus early years programme: A video-based early intervention for parents of pre-school children with behavioural and developmental difficulties. *Clinical Child Psychology and Psychiatry*,*10*(3), 319-336. [https://doi.org/https://dx.doi.org/10.1177/1359104505053752](https://doi.org/https:/dx.doi.org/10.1177/1359104505053752)

Siegel, M., Milligan, B., Chemelski, B., Payne, D., Ellsworth, B., Harmon, J., Teer, O., & Smith, K. A. (2014). Specialized inpatient psychiatry for serious behavioral disturbance in autism and intellectual disability. *Journal of Autism and Developmental Disorders*,*44*(12), 3026-3032. [https://doi.org/https://dx.doi.org/10.1007/s10803-014-2157-z](https://doi.org/https:/dx.doi.org/10.1007/s10803-014-2157-z)

Skoien-Bradley, R. A. (1992). The effects of parental involvement with preschoolers at risk for developmental and behavioral problems. *Dissertation Abstracts International Section A: Humanities and Social Sciences*,*52*(8), 2867-2868.

Smith, T., Eikeseth, S., Klevstr, M., & Lovaas, O. I. (1997). Intensive behavioral treatment for preschoolers with severe mental retardation and pervasive developmental disorder. *American journal of mental retardation : AJMR*,*102*(3), 238-249.

Smith, T., Eikeseth, S., Klevstr, M., & Lovaas, O. I. (1997). Intensive behavioral treatment for preschoolers with severe mental retardations and pervasive developmental disorder. *American Journal on Mental Retardation*,*102*(3), 238-249. [https://doi.org/https://dx.doi.org/10.1352/0895-8017%281997%29102%3C0238:IBTFPW%3E2.0.CO;2](https://doi.org/https:/dx.doi.org/10.1352/0895-8017%281997%29102%3C0238:IBTFPW%3E2.0.CO;2)

Sofronoff, K., Jahnel, D., S, M., & ers. (2011). Stepping Stones Triple P seminars for parents of a child with a disability: A randomized controlled trial. *Research in developmental disabilities*,*32*(6), 2253-2262. [https://doi.org/https://dx.doi.org/10.1016/j.ridd.2011.07.046](https://doi.org/https:/dx.doi.org/10.1016/j.ridd.2011.07.046)

Spijkers, W., Jansen, D. E., & Reijneveld, S. A. (2013). Effectiveness of Primary Care Triple P on child psychosocial problems in preventive child healthcare: A randomized controlled trial. *BMC Med*,*11*, 240. <https://doi.org/10.1186/1741-7015-11-240>

Steeger, C. M. (2014). Combined cognitive and parent training interventions for adolescents with ADHD and their mothers: A randomized, controlled trial. *Child Neuropsychology*,*22*(4), 394-419.

Stuttard, L., Beresford, B., Clarke, S., Beecham, J., Todd, S., & Bromley, J. (2014). Riding the rapids: living with autism or disability--an evaluation of a parenting support intervention for parents of disabled children. *Research in developmental disabilities*,*35*(10), 2371-2383. [https://doi.org/https://dx.doi.org/10.1016/j.ridd.2014.05.021](https://doi.org/https:/dx.doi.org/10.1016/j.ridd.2014.05.021)

Talkington, L., & Watters, L. (1970). Programming for special class misfits. *Mental Retardation*,*8*(2), 27-29.

Tavormina, J. B. (1975). Relative effectiveness of behavioral and reflective group counseling with parents of mentally retarded children. *Journal of Consulting and Clinical Psychology*,*43*(1), 22-31. [https://doi.org/https://dx.doi.org/10.1037/h0076328](https://doi.org/https:/dx.doi.org/10.1037/h0076328)

Thomeer, M. L., Lopata, C., Rodgers, J. D., Donnelly, J. P., Jordan, A. K., Booth, A. J., & McDonald, C. A. (2020). Feasibility and initial efficacy of a cognitive-behavioral summer treatment for young children with ASD. *Journal of Developmental and Physical Disabilities*,*32*(5), 735-754. [https://doi.org/https://dx.doi.org/10.1007/s10882-019-09717-w](https://doi.org/https:/dx.doi.org/10.1007/s10882-019-09717-w)

To, M. Y., & Chan, S. (2000). Evaluating the effectiveness of progressive muscle relaxation in reducing the aggressive behaviors of mentally handicapped patients. *Archives of psychiatric nursing*,*14*(1), 39-46.

Turygin, N., Matson, J. L., Williams, L. W., & Belva, B. C. (2014). The relationship of parental first concerns and autism spectrum disorder in an early intervention sample. *Research in Autism Spectrum Disorders*,*8*(2), 53-60. [https://doi.org/https://dx.doi.org/10.1016/j.rasd.2013.10.008](https://doi.org/https:/dx.doi.org/10.1016/j.rasd.2013.10.008)

Visscher, L., Reijneveld, S. A., Knot-Dickscheit, J., Yperen, T. A. v., Scholte, R. H. J., Delsing, M. J. M. H., Evenboer, K. E., & Jansen, D. E. M. C. (2022). Toward tailored care for families with multiple problems: A quasi-experimental study on effective elements of care. *Family process*,*61*(2), 571-590. [https://doi.org/https://dx.doi.org/10.1111/famp.12745](https://doi.org/https:/dx.doi.org/10.1111/famp.12745)

White, G. D., Nielsen, G., & Johnson, S. M. (1972). Timeout duration and the suppression of deviant behavior in children. *Journal of Applied Behavior Analysis*,*5*(2), 111-120. [https://doi.org/https://dx.doi.org/10.1901/jaba.1972.5-111](https://doi.org/https:/dx.doi.org/10.1901/jaba.1972.5-111)

Zimmerman, F. T., & Burgemeister, B. B. (1955). Preliminary report upon the effect of reserpine on epilepsy and behavior problems in children. *Annals of the New York Academy of Sciences*,*61*(1), 215-221.

**Sample too old**

Akefeldt, A., & Gillberg, C. (1999). Behavior and personality characteristics of children and young adults with Prader-Willi syndrome: A controlled study. *Journal of the American Academy of Child & Adolescent Psychiatry*,*38*(6), 761-769. [https://doi.org/https://dx.doi.org/10.1097/00004583-199906000-00025](https://doi.org/https:/dx.doi.org/10.1097/00004583-199906000-00025)

Asmus, J. M., Ringdahl, J. E., Sellers, J. A., Call, N. A., S., M., elman, & Wacker, D. P. (2004). Use of a short-term inpatient model to evaluate aberrant behavior: Outcome data summaries from 1996 to 2001. *Journal of Applied Behavior Analysis*,*37*(3), 283-304. [https://doi.org/https://dx.doi.org/10.1901/jaba.2004.37-283](https://doi.org/https:/dx.doi.org/10.1901/jaba.2004.37-283)

Bowring, D. L., Totsika, V., Hastings, R. P., & Toogood, S. (2020). Outcomes from a community-based positive behavioural support team for children and adults with developmental disabilities. *Journal of Applied Research in Intellectual Disabilities*,*33*(2), 193-203.

Chan, S., Fung, M. Y., Tong, C. W., & Thompson, D. (2005). The clinical effectiveness of a multisensory therapy on clients with developmental disability. *Research in developmental disabilities*,*26*(2), 131-142.

Eratay, E. (2013). Effectiveness of leisure time activities program on social skills and behavioral problems in individuals with intellectual disabilities. *Educational Research and Reviews*,*8*(16), 1437-1448.

Gerber, F., Bessero, S., Robbiani, B., Courvoisier, D. S., Baud, M. A., Traore, M. C., Blanco, P., Giroud, M., & Carminati, G. G. (2011). Comparing residential programmes for adults with autism spectrum disorders and intellectual disability: Outcomes of challenging behaviour and quality of life. *Journal of Intellectual Disability Research*,*55*(9), 918-932.

Hulsmans, D. H. G., Otten, R., Schijven, E. P., & Poelen, E. A. P. (2021). Exploring the role of emotional and behavioral problems in a personality-targeted prevention program for substance use in adolescents and young adults with intellectual disability. *Research in developmental disabilities*,*109*. [https://doi.org/https://dx.doi.org/10.1016/j.ridd.2020.103832](https://doi.org/https:/dx.doi.org/10.1016/j.ridd.2020.103832)

Hundert, J. (1997). The effectiveness of a three-session, brief behavioral consultation for caregivers of children and adults with developmental disabilities. *Journal on Developmental Disabilities*,*5*(1), 24-46.

MacDonald, A., McGill, P., & Murphy, G. (2018). An evaluation of staff training in positive behavioural support. *Journal of applied research in intellectual disabilities : JARID*,*31*(6), 1046-1061. [https://doi.org/https://dx.doi.org/10.1111/jar.12460](https://doi.org/https:/dx.doi.org/10.1111/jar.12460)

Mildon, R., Wade, C., & Matthews, J. (2008). Considering the contextual fit of an intervention for families headed by parents with an intellectual disability: An exploratory study. *Journal of Applied Research in Intellectual Disabilities*,*21*(4), 377-387. [https://doi.org/https://dx.doi.org/10.1111/j.1468-3148.2008.00451.x](https://doi.org/https:/dx.doi.org/10.1111/j.1468-3148.2008.00451.x)

Rose, J., Loftus, M., Flint, B., & Carey, L. (2005). Factors associated with the efficacy of a group intervention for anger in people with intellectual disabilities. *The British journal of clinical psychology*,*44*, 305-317.

Steffy, R. A. (1970). Behavior therapy with regressed, aggressive psychiatric patients. *Current psychiatric therapies*,*10*, 191-202.

Tsang, B., Leung, C. N. W., & Chan, R. W. S. (2022). A feasibility study on social competence intervention for chinese adolescents and adults with comorbid autism spectrum disorder and intellectual disability. *Journal of Applied Research in Intellectual Disabilities*,*35*(5), 1131-1139.

**Not psychosocial intervention**

Beh-Pajooh, A., Abdollahi, A., & Hosseinian, S. (2018). The effectiveness of painting therapy program for the treatment of externalizing behaviors in children with intellectual disability. *Vulnerable Children and Youth Studies*,*13*(3), 221-227. [https://doi.org/https://dx.doi.org/10.1080/17450128.2018.1428779](https://doi.org/https:/dx.doi.org/10.1080/17450128.2018.1428779)

Benda, C. E., Squires, N. D., Ogonik, J., Wise, R., & Akin, R. (1964). The relationship between intellectual inadequacy and emotional and sociocultural privation. *Comprehensive psychiatry*,*5*, 294-313.

Bull, L. E., Oliver, C., Callaghan, E., & Woodcock, K. A. (2015). Increased exposure to rigid routines can lead to increased challenging behavior following changes to those routines. *J Autism Dev Disord*,*45*(6), 1569-1578. <https://doi.org/10.1007/s10803-014-2308-2>

Epp, K. M. (2008). Outcome-based evaluation of a social skills program using art therapy and group therapy for children on the autism spectrum. *Children & Schools*,*30*(1), 27-36.

Greene, R. J., & Pratt, J. J. (1972). A group contingency for individual misbehaviors in the classroom. *Mental Retardation*,*10*(3), 33-35.

Hudson, A., Wilken, P., Jauernig, R., & Radler, G. (1995). Regionally based teams for the treatment of challenging behaviour: A three-year outcome study. *Behaviour Change*,*12*(4), 209-215.

Kirk, H., Gray, K., Ellis, K., Taffe, J., & Cornish, K. (2017). Impact of attention training on academic achievement, executive functioning, and behavior: A randomized controlled trial. *American Journal on Intellectual and Developmental Disabilities*,*122*(2), 97-117.

LaMont, M. (2011). Mother-child attachment and preschool behavior problems in children with developmental delay. *ProQuest LLC*.

Laveck, G. D., Cruz, F. D. L., & Simundson, E. (1960). Fluphenazine in the treatment of mentally retarded children with behavior disorders. *Diseases of the nervous system*,*21*, 82-85.

Lindgren, S., Wacker, D., Suess, A., Schieltz, K., Pelzel, K., Kopelman, T., Lee, J., Romani, P., & Waldron, D. (2016). Telehealth and autism: Treating challenging behavior at lower cost. *Pediatrics*,*137*, S167-S175. [https://doi.org/https://dx.doi.org/10.1542/peds.2015-2851O](https://doi.org/https:/dx.doi.org/10.1542/peds.2015-2851O)

R. J. Thompson, J. (1985). Delineation of children's behavior problems: a basis for assessment and intervention. *Journal of developmental and behavioral pediatrics : JDBP*,*6*(1), 37-50.

Reardon, D. F., Butler, K., & Warshaw, K. (1978). The effect of an unlocked door on adolescent runaway and aggression. *Journal of the American Academy of Child Psychiatry*,*17*(2), 372-382.

Rivard, M., Morin, D., Dionne, C., Mello, C., & Gagnon, M.-A. (2015). Assessment, intervention, and training needs of service providers for children with intellectual disabilities or autism spectrum disorders and concurrent problem behaviours. *Exceptionality Education International*,*25*(2), 65-83.

Ryan, S., Lai, J., & Weiss, J. A. (2018). Mental health service use among youth with autism spectrum disorder: A comparison of two age groups. *Journal of Developmental and Behavioral Pediatrics*,*39*(1), 20-27.

Ulrey, G., Hudler, M., Marshall, R., Wuori, D., & Cranston, C. (1987). A community model for physician, educator, and parent collaboration for management of children with developmental and behavioral disorders. *Special Issue: Developmental and behavioral disorders*,*26*(5), 235-239. [https://doi.org/https://dx.doi.org/10.1177/000992288702600504](https://doi.org/https:/dx.doi.org/10.1177/000992288702600504)

Wortis, J., Floistad, I., Sersen, E. A., & Astrup, C. (1973). Childhood and adult behavior disorder: some experimental comparisons. *Conditional reflex*,*8*(2), 88-97.

**Different intervention aim**

Achtergarde, S., Becke, J., Beyer, T., Postert, C., Romer, G., & Muller, J. M. (2014). Preschool-age male psychiatric patients with specific developmental disorders and those without: Do they differ in behavior problems and treatment outcome? *Infants & Young Children*,*27*(4), 359-377. [https://doi.org/https://dx.doi.org/10.1097/IYC.0000000000000020](https://doi.org/https:/dx.doi.org/10.1097/IYC.0000000000000020)

Albini, J. L., & Dinitz, S. (1965). Psychotherapy with disturbed and defective children: an evaluation of changes in behavior and attitudes. *American journal of mental deficiency*,*69*, 560-567.

Cohen, N. J., Bradley, S., & Kolers, N. (1987). Outcome evaluation of a therapeutic day treatment program for delayed and disturbed preschoolers. *Journal of the American Academy of Child & Adolescent Psychiatry*,*26*(5), 687-693. [https://doi.org/https://dx.doi.org/10.1097/00004583-198709000-00012](https://doi.org/https:/dx.doi.org/10.1097/00004583-198709000-00012)

Coles, C. D., Strickl, D. C., Padgett, L., & Bellmoff, L. (2007). Games that work: Using computer games to teach alcohol-affected children about fire and street safety. *Research in developmental disabilities*,*28*(5), 518-530. [https://doi.org/https://dx.doi.org/10.1016/j.ridd.2006.07.001](https://doi.org/https:/dx.doi.org/10.1016/j.ridd.2006.07.001)

Davis, H., & Rushton, R. (1991). Counselling and supporting parents of children with developmental delay: A research evaluation. *Journal of Mental Deficiency Research*,*35*(2), 89-112.

Gottwald, H. L. (1964). A special program for educable--emotionally disturbed retarded. *Mental Retardation*,*2*(6), 353-359.

Hall, S. S., Rodriguez, A. B., Jo, B., & Pollard, J. S. (2022). Long-term follow-up of telehealth-enabled behavioral treatment for challenging behaviors in boys with fragile X syndrome. *Journal of Neurodevelopmental Disorders*,*14*. [https://doi.org/https://dx.doi.org/10.1186/s11689-022-09463-9](https://doi.org/https:/dx.doi.org/10.1186/s11689-022-09463-9)

Hauser-Cram, P., Warfield, M. E., Shonkoff, J. P., Krauss, M. W., Sayer, A., & Upshur, C. C. (2001). Children with disabilities: a longitudinal study of child development and parent well-being. *Monogr Soc Res Child Dev*,*66*(3), i-viii, 1-114; discussion 115-126.

Kotsopoulos, S. I., Karaivazoglou, K., Florou, I. S., Gyftogianni, M. I., Papadaki, E. J., & Kotsopoulou, A. (2021). Systematic intervention for children with autism spectrum disorder and integration in regular school classes: A naturalistic study. *Global Pediatric Health*,*8*. <https://doi.org/10.1177/2333794X211012988>

Lee, M.-Y., & Gaucher, R. (2000). Group treatment for dually diagnosed adolescents: An empowerment-based approach. *Social Work with Groups*,*23*(2), 55-78. [https://doi.org/https://dx.doi.org/10.1300/J009v23n02_05](https://doi.org/https:/dx.doi.org/10.1300/J009v23n02_05)

Leong, H. M., Stephenson, J., & Carter, M. (2014). The use of sensory integration therapy in malaysia and singapore by special education teachers in early intervention settings. *Journal of Intellectual & Developmental Disability*,*39*(1), 10-23.

Nestler, J., & Goldbeck, L. (2011). A pilot study of social competence group training for adolescents with borderline intellectual functioning and emotional and behavioural problems (SCT-ABI). *Journal of Intellectual Disability Research*,*55*(2), 231-241. [https://doi.org/https://dx.doi.org/10.1111/j.1365-2788.2010.01369.x](https://doi.org/https:/dx.doi.org/10.1111/j.1365-2788.2010.01369.x)

Pears, K. C., Kim, H. K., Healey, C. V., Yoerger, K., & Fisher, P. A. (2015). Improving child self-regulation and parenting in families of pre-kindergarten children with developmental disabilities and behavioral difficulties. *Prevention Science*,*16*(2), 222-232. [https://doi.org/https://dx.doi.org/10.1007/s11121-014-0482-2](https://doi.org/https:/dx.doi.org/10.1007/s11121-014-0482-2)

Ratcliffe, B., Wong, M., Dossetor, D., & Hayes, S. (2019). Improving emotional competence in children with autism spectrum disorder and mild intellectual disability in schools: A preliminary treatment versus waitlist study. *Behaviour Change*,*36*(4), 216-232. <https://doi.org/10.1017/bec.2019.13>

Riemersma, I., Santvoort, F. V., Doesum, K. T. M. V., Hosman, C. M. H., Janssens, J. M. A. M., Z, R. A. P. V. d., en, & Otten, R. (2022). 'You are Okay': Effects of a support and educational program for children with mild intellectual disability and their parents with mental health concerns. *Journal of intellectual disabilities : JOID*,*26*(1), 70-89. [https://doi.org/https://dx.doi.org/10.1177/1744629520953765](https://doi.org/https:/dx.doi.org/10.1177/1744629520953765)

Smith, T., Groen, A. D., & Wynn, J. W. (2000). Randomized trial of intensive early intervention for children with pervasive developmental disorder. *American Journal on Mental Retardation*,*105*(4), 269-285. [https://doi.org/https://dx.doi.org/10.1352/0895-8017%282000%29105%3C0269:RTOIEI%3E2.0.CO;2](https://doi.org/https:/dx.doi.org/10.1352/0895-8017%282000%29105%3C0269:RTOIEI%3E2.0.CO;2)

Verberg, F., Helmond, P., Otten, R., & Overbeek, G. (2022). The online mindset intervention 'The Growth Factory' for adolescents with intellectual disabilities: moderators and mediators. *Journal of intellectual disability research : JIDR*,*66*(10), 817-832. [https://doi.org/https://dx.doi.org/10.1111/jir.12970](https://doi.org/https:/dx.doi.org/10.1111/jir.12970)

Whitehead, R., Hopkins, L., Hughes, E., Kehoe, M., & Pedwell, G. (2021). Everyone on the same team, all working together: Implementing a co-ordinated multi-disciplinary approach to supporting young people with co-occurring intellectual disability and mental health issues. *Journal of Mental Health Research in Intellectual Disabilities*,*14*(1), 1-22.

**Lack of pre-post externalising problems measure**

Feldman, M. A., & Werner, S. E. (2002). Collateral effects of behavioral parent training on families of children with developmental disabilities and behavior disorders. *Behavioral Interventions*,*17*(2), 75-83. [https://doi.org/https://dx.doi.org/10.1002/bin.111](https://doi.org/https:/dx.doi.org/10.1002/bin.111)

Fleming, E. R., & Fleming, D. C. (1982). Social skill training for educable mentally retarded children. *Education & Training of the Mentally Retarded*,*17*(1), 44-50.

Hall, S. S., Monlux, K. D., Rodriguez, A. B., Jo, B., & Pollard, J. S. (2020). Telehealth-enabled behavioral treatment for problem behaviors in boys with fragile X syndrome: a randomized controlled trial. *Journal of Neurodevelopmental Disorders*,*12*(1), 31. [https://doi.org/https://dx.doi.org/10.1186/s11689-020-09331-4](https://doi.org/https:/dx.doi.org/10.1186/s11689-020-09331-4)

Kodra, Y., Kondili, L. A., Ferraroni, A., Serra, M. A., Caretto, F., Ricci, M. A., & Taruscio, D. (2016). Parent training education program: a pilot study, involving families of children with Prader-Willi syndrome. *Annali dell'Istituto superiore di sanita*,*52*(3), 428-433. <https://doi.org/https:/dx.doi.org/10.4415/ANN_16_03_15>

McIntyre, L. L., Neece, C. L., Sanner, C. M., Rodriguez, G., & Safer-Lichtenstein, J. (2022). Telehealth delivery of a behavioral parent training program to Spanish-speaking Latinx parents of young children with developmental delay: Applying an implementation framework approach. *School Psychology Review*,*51*(2), 206-220. [https://doi.org/https://dx.doi.org/10.1080/2372966X.2021.1902749](https://doi.org/https:/dx.doi.org/10.1080/2372966X.2021.1902749)

Miezio, S. (1967). Group therapy with mentally retarded adolescents in institutional settings. *International Journal of Group Psychotherapy*,*17*(3), 321-327.

Monlux, K. D., Pollard, J. S., Buj, A. Y., Rodriguez, a., & Hall, S. S. (2019). Telehealth delivery of function-based behavioral treatment for problem behaviors exhibited by boys with Fragile X Syndrome. *Journal of Autism and Developmental Disorders*,*49*(6), 2461-2475.

Scahill, L., Bearss, K., Lecavalier, L., Smith, T., Swiezy, N., Aman, M. G., Sukhodolsky, D. G., McCracken, C., Minshawi, N., Turner, K., Levato, L., Saulnier, C., Dziura, J., & Johnson, C. (2016). Effect of parent training on adaptive behavior in children with autism spectrum disorder and disruptive behavior: Results of a randomized trial. *Journal of the American Academy of Child & Adolescent Psychiatry*,*55*(7), 602-609. [https://doi.org/https://dx.doi.org/10.1016/j.jaac.2016.05.001](https://doi.org/https:/dx.doi.org/10.1016/j.jaac.2016.05.001)

Viola, T. (2007). Remediating behaviour problems in children with developmental disabilities. *Dissertation Abstracts International Section A: Humanities and Social Sciences*,*68*(3), 878.

Wacker, D. P., Berg, W. K., Harding, J. W., Barretto, A., Rankin, B., & Ganzer, J. (2005). Treatment effectiveness, stimulus generalization, and acceptability to parents of functional communication training. *Educational Psychology*,*25*(2), 233-256. [https://doi.org/https://dx.doi.org/10.1080/0144341042000301184](https://doi.org/https:/dx.doi.org/10.1080/0144341042000301184)

**Not English language**

Ilg, J., Jebrane, A., Dutray, B., Wolgensinger, L., Rousseau, M., Paquet, A., & Clément, C. (2017). Evaluation of a French parent-training program in young children with autism spectrum disorder: A pilot study. *Annales Medico-Psychologiques*,*175*(5), 430-435. <https://doi.org/10.1016/j.amp.2016.01.018>

# S7. Table: Risk of bias evaluation

|  | | | | | | |
| --- | --- | --- | --- | --- | --- | --- |
| **Study** | **D1** | **D2** | **D3** | **D4** | **D5** | **Overall** |
| Acosta et al. (2019) |  |  |  |  |  |  |
| Blankestein et al. (2019) |  |  |  |  |  |  |
| Te Brinke et al. (2022) |  |  |  |  |  |  |
| Hand et al. (2013) |  |  |  |  |  |  |
| Lakhan (2014) |  |  |  |  |  |  |
| McMahon et al. (2023) |  |  |  |  |  |  |
| Schuiringa et al. (2017) |  |  |  |  |  |  |
| *Note*. D1 = randomisation process, D2 = deviations from the intended interventions, D3 = missing outcome data, D4 = measurement of the outcome, D5 = selection of the reported result, Overall = overall risk of bias rating of the study, + = low risk, ! = some concerns, − = high risk. | | | | | | |

# S8. Figure: Sensitivity analysis funnel plot with standard errors along the Y-Axis plotted against effect controlled and pre- to post-test sizes (Hedge’s g) along the X-Axis, after removal of the outlier


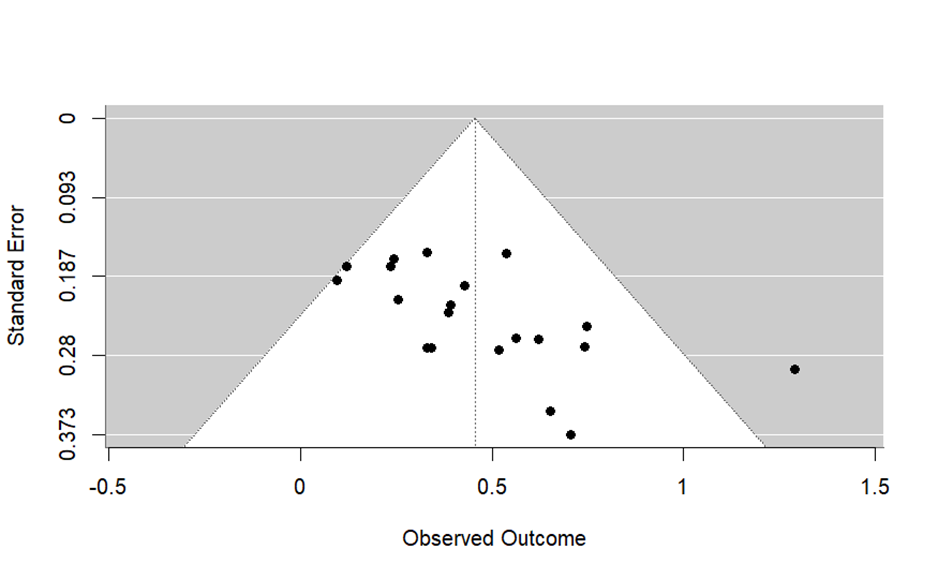


# S9. PRISMA checklist

| **Section and Topic** | **Item #** | **Checklist item** | **Location where item is reported** |
| --- | --- | --- | --- |
| **TITLE** | | |  |
| Title | 1 | Identify the report as a systematic review. | p. 1 |
| **ABSTRACT** | | |  |
| Abstract | 2 | See the PRISMA 2020 for Abstracts checklist. | 3 |
| **INTRODUCTION** | | |  |
| Rationale | 3 | Describe the rationale for the review in the context of existing knowledge. | pp. 4-8 |
| Objectives | 4 | Provide an explicit statement of the objective(s) or question(s) the review addresses. | 8 |
| **METHODS** | | |  |
| Eligibility criteria | 5 | Specify the inclusion and exclusion criteria for the review and how studies were grouped for the syntheses. | pp. 8-9 |
| Information sources | 6 | Specify all databases, registers, websites, organisations, reference lists and other sources searched or consulted to identify studies. Specify the date when each source was last searched or consulted. | p. 9, S3 |
| Search strategy | 7 | Present the full search strategies for all databases, registers and websites, including any filters and limits used. | S2 |
| Selection process | 8 | Specify the methods used to decide whether a study met the inclusion criteria of the review, including how many reviewers screened each record and each report retrieved, whether they worked independently, and if applicable, details of automation tools used in the process. | p. 9, S3 |
| Data collection process | 9 | Specify the methods used to collect data from reports, including how many reviewers collected data from each report, whether they worked independently, any processes for obtaining or confirming data from study investigators, and if applicable, details of automation tools used in the process. | p. 9, S3 |
| Data items | 10a | List and define all outcomes for which data were sought. Specify whether all results that were compatible with each outcome domain in each study were sought (e.g. for all measures, time points, analyses), and if not, the methods used to decide which results to collect. | pp. 9-10 |
|  | 10b | List and define all other variables for which data were sought (e.g. participant and intervention characteristics, funding sources). Describe any assumptions made about any missing or unclear information. | p. 10 |
| Study risk of bias assessment | 11 | Specify the methods used to assess risk of bias in the included studies, including details of the tool(s) used, how many reviewers assessed each study and whether they worked independently, and if applicable, details of automation tools used in the process. | p. 10-11 |
| Effect measures | 12 | Specify for each outcome the effect measure(s) (e.g. risk ratio, mean difference) used in the synthesis or presentation of results. | p. 11 |
| Synthesis methods | 13a | Describe the processes used to decide which studies were eligible for each synthesis (e.g. tabulating the study intervention characteristics and comparing against the planned groups for each synthesis (item #5)). | p. 11-12 |
|  | 13b | Describe any methods required to prepare the data for presentation or synthesis, such as handling of missing summary statistics, or data conversions. | p. 12, S3 |
|  | 13c | Describe any methods used to tabulate or visually display results of individual studies and syntheses. | p. 13 |
|  | 13d | Describe any methods used to synthesize results and provide a rationale for the choice(s). If meta-analysis was performed, describe the model(s), method(s) to identify the presence and extent of statistical heterogeneity, and software package(s) used. | pp. 12-14 |
|  | 13e | Describe any methods used to explore possible causes of heterogeneity among study results (e.g. subgroup analysis, meta-regression). | pp. 13-14 |
|  | 13f | Describe any sensitivity analyses conducted to assess robustness of the synthesized results. | p. 12 |
| Reporting bias assessment | 14 | Describe any methods used to assess risk of bias due to missing results in a synthesis (arising from reporting biases). | p. 14 |
| Certainty assessment | 15 | Describe any methods used to assess certainty (or confidence) in the body of evidence for an outcome. | NA |
| **RESULTS** | | |  |
| Study selection | 16a | Describe the results of the search and selection process, from the number of records identified in the search to the number of studies included in the review, ideally using a flow diagram. | p. 14, Figure 1 |
|  | 16b | Cite studies that might appear to meet the inclusion criteria, but which were excluded, and explain why they were excluded. | S4, S5 |
| Study characteristics | 17 | Cite each included study and present its characteristics. | Table 1 |
| Risk of bias in studies | 18 | Present assessments of risk of bias for each included study. | p. 16, S6 |
| Results of individual studies | 19 | For all outcomes, present, for each study: (a) summary statistics for each group (where appropriate) and (b) an effect estimate and its precision (e.g. confidence/credible interval), ideally using structured tables or plots. | Figure 2 |
| Results of syntheses | 20a | For each synthesis, briefly summarise the characteristics and risk of bias among contributing studies. | pp. 14-16 |
|  | 20b | Present results of all statistical syntheses conducted. If meta-analysis was done, present for each the summary estimate and its precision (e.g. confidence/credible interval) and measures of statistical heterogeneity. If comparing groups, describe the direction of the effect. | pp. 16-17 |
|  | 20c | Present results of all investigations of possible causes of heterogeneity among study results. | pp. 17-18 |
|  | 20d | Present results of all sensitivity analyses conducted to assess the robustness of the synthesized results. | pp. 18 |
| Reporting biases | 21 | Present assessments of risk of bias due to missing results (arising from reporting biases) for each synthesis assessed. | pp. 18 |
| Certainty of evidence | 22 | Present assessments of certainty (or confidence) in the body of evidence for each outcome assessed. | NA |
| **DISCUSSION** | | |  |
| Discussion | 23a | Provide a general interpretation of the results in the context of other evidence. | pp. 19-21 |
|  | 23b | Discuss any limitations of the evidence included in the review. | p. 23 |
|  | 23c | Discuss any limitations of the review processes used. | p. 23 |
|  | 23d | Discuss implications of the results for practice, policy, and future research. | pp. 21-24 |
| **OTHER INFORMATION** | | |  |
| Registration and protocol | 24a | Provide registration information for the review, including register name and registration number, or state that the review was not registered. | p. 8 |
|  | 24b | Indicate where the review protocol can be accessed, or state that a protocol was not prepared. | p. 8 |
|  | 24c | Describe and explain any amendments to information provided at registration or in the protocol. | NA |
| Support | 25 | Describe sources of financial or non-financial support for the review, and the role of the funders or sponsors in the review. | p. 25 |
| Competing interests | 26 | Declare any competing interests of review authors. | p. 25 |
| Availability of data, code and other materials | 27 | Report which of the following are publicly available and where they can be found: template data collection forms; data extracted from included studies; data used for all analyses; analytic code; any other materials used in the review. | p. 8 |
